# Expression of functional human sialyltransferases ST6GalNAc5 and ST6GalNAc6 in *Pichia pastoris*

**DOI:** 10.1007/s00253-025-13607-x

**Published:** 2025-10-15

**Authors:** Ganna Krasnoselska, Marton Lengyel, Martin Matwiejuk, Marlene Vuillemin, Dora Molnar-Gabor, Anne S. Meyer, Birgitte Zeuner

**Affiliations:** 1 Department of Biotechnology and Biomedicine, Technical University of Denmark, Søltofts Plads 221, 2800 Kgs. Lyngby, Denmark; 2 DSM-Firmenich, Kogle Allé 4, 2970 Hørsholm, Denmark

**Keywords:** Sialyltransferase, Disialyllacto-*N*-tetraose, *N*-Glycosylation, *Pichia pastoris*, Protein engineering

## Abstract

**Abstract:**

The two sialyltransferases in the ST6GALNAC subfamily (EC 2.4.99.-; CAZy family GT29), ST6GalNAc5 and ST6GalNAc6, catalyze the formation of the linkage from the sialic acid moiety to the C6 position of *N*-acetylgalactosamine (GalNAc) as well as to *N*-acetylglucosamine (GlcNAc), and are known as α-2,6-sialyltransferases. This activity is interesting for the synthesis of the disialylated oligosaccharide disialyllacto-*N*-tetraose (DSLNT). Human sialyltransferases ST6GalNAc5 and ST6GalNAc6 produced in HEK293 cells are commercially available at a smaller scale. In this study, we demonstrated that ST6GalNAc5 and ST6GalNAc6 can be functionally expressed in *Pichia pastoris* X-33. The level of ST6GalNAc5 and ST6GalNAc6 expression and activity largely depended on the type of construct, as well as on expression conditions, namely temperature, methanol feeding regime, and supplements. Insertion of a (GGGS)₂ linker peptide between the gene and the α secretion factor improved the secretion of active enzyme in *P. pastoris* X-33. The use of media supplemented with MgCl_2_ and Casamino acids led to increased cell growth and, importantly, enhanced ST6GalNAc5 and ST6GalNAc6 production. Under optimized conditions, the *P. pastoris* X-33 strain could secrete up to 10 mg of active sialyltransferase protein per liter of culture. Compared to their wild-type counterparts, mutants of ST6GalNAc5 and ST6GalNAc6 devoid of *N*-glycosylation sites exhibited reduced enzymatic activity and stability. Apart from contributing to successful *P. pastoris* expression, our findings also contribute to a deeper understanding of the role of *N*-glycosylation in the activity and stability of sialyltransferases.

**Key points:**

• *Expression of functional human ST6GalNAc5 and ST6GalNAc6 in Pichia pastoris*

• *Mutants devoid of N-glycosylations lack activity*

• *Media supplementation with MgCl2 and Casamino acids improves expression*

**Supplementary Information:**

The online version contains supplementary material available at 10.1007/s00253-025-13607-x.

## Introduction

The biosynthesis of sialylconjugates in mammals is mediated by a specific family of glycosyltransferases, EC 2.4.99.-, from the GT29 family of the CAZy database known as sialyltransferases (STs) (Harduin-Lepers 2010). Currently, 20 human ST genes are known. The STs utilize a cytidine monophosphate (CMP)–activated donor substrate, such as CMP-*N*-acetylneuraminic acid (CMP-Neu5Ac), and catalyze the sialylation of various acceptor substrates, including glycoconjugates containing galactose, *N*-acetylgalactosamine (GalNAc), or sialic acid.

Among the four ST subfamilies, the *N*-acetylgalactosaminide α−2,6-sialyltransferase (ST6GalNAc) subfamily specifically catalyzes the transfer of a sialic acid moiety onto a GalNAc residue to form a α−2,6-linkage (Harduin-Lepers et al. 1995; Harduin-Lepers et al. 2001). In mammals, there are six ST6GalNAc proteins, named ST6GalNAc1-6. Of these, ST6GalNAc5-6 are believed to also accept GlcNAc as their acceptor residue (Harduin-Lepers 2023). The full-length ST6GalNAc5 and ST6GalNAc6 are transmembrane, Golgi-localized enzymes. ST6GalNAc5 is predominantly expressed in adult brain tissue, and ST6GalNAc6 is more broadly expressed in nervous tissue as well as in various other organs, including the colon, rectum, pancreas, lungs, liver, and heart. Both enzymes have been reported to function in the production of α-series gangliosides, specifically in the biosynthesis of ganglioside GD1α from GM1b through the α2,6-linked attachment of Neu5Ac to the GalNAc residue (Harduin-Lepers 2013). Recently, these enzymes have gained additional attention in the production of the (di)sialylated human milk oligosaccharides (HMOs) sialyllacto-*N*-tetraose b (LST-b) and disialyllacto-*N*-tetraose (DSLNT) (Bao et al., 2024). It is not fully elucidated which of the ST6GALNACs are responsible for the biosynthesis of these HMOs in the human mammary gland (Kellman et al., 2022); yet recent in vitro demonstration points towards ST6GALNAC4-6 as the main candidates (Bao et al., 2024).

The HMOs are the third largest component of human milk and comprise over 200 nondigestible, structurally diverse carbohydrates built on a lactose core, which through various glycosidic linkages is decorated with fucose and/or the sialic acid Neu5Ac and elongated with lacto-*N*-biose and/or *N*-acetyllactosamine (Bode 2012; Hill et al., 2021). The sialylated HMOs, which constitute 10–30% of total HMOs in milk, serve as a prebiotic for probiotic bacteria in the gut environment and as an essential component for newborn brain development (Viverge et al., 1990; Coppa et al., 1993; Yu et al., 2013; Bode, 2015). Low concentrations of DSNLT in the milk have been associated with the development of necrotizing enterocolitis (NEC) in preterm infants (Masi et al., 2021; Autran et al., 2018). Further, DSLNT has been observed to protect rat models from NEC, which is a common intestinal disorder in preterm infants with a very low birth rate and has a high mortality rate (Bode 2018). The beneficial effects of DSLNT provide a strong incentive for aiming at producing this HMO via enzymatic synthesis.

There are only a few reports on the successful recombinant expression of ST6GalNAc5 and ST6GalNAc6. Given their eukaryotic origin, both enzymes have been successfully produced in eukaryotic hosts such as HEK293 cells and the baculovirus-insect cell system (Moremen et al., 2018; Bao et al., 2024). By truncating 49 amino acids in ST6GalNAc5 and 30 in ST6GalNAc6, Moremen et al. (2018) achieved approximately 50% secretion efficiency in HEK293 cells, though partial proteolysis was observed. The reported yield for the secreted protein was around 40 mg/L. Building on this strategy, Bao et al. (2024) successfully expressed functional Δ29ST6GalNAc6 and compared its activity profile with the now commercially available Δ49ST6GalNAc5 produced in HEK293 cells, reporting appreciable activity of both enzymes for DSLNT synthesis (Bao et al., 2024).

In 2024, Pei et al. supplemented *Escherichia coli* BL21(DE3) and Origami 2(DE3) with additional chaperones (e.g., pGro7) to improve the folding and stability of Δ50ST6GalNAc5 and Δ64ST6GalNAc6 in the first report of microbial expression of these STs (Pei et al. 2024). While they successfully produced these proteins, the enzymatic activity and substrate specificity were significantly lower compared to enzymes expressed in mammalian cells, highlighting the limitations of bacterial expression systems for complex eukaryotic glycosyltransferases. Recently, Bai et al. found that the addition of an N-terminal maltose binding protein (MBP) domain to Δ49ST6GalNAc5 in a construct with a C-terminal His_6_ tag improved expression and protein stability (Bai et al., 2025).

While another human ST, ST6Gal1, has been expressed in *Pichia pastoris* (Ribitsch et al. 2014), there have been no reports of successful recombinant expression of ST6GalNAc5 or ST6GalNAc6 in yeast, despite the advantages yeast systems offer. Yeast expression platforms can promote correct disulfide bond formation, support N-linked glycosylation, and mitigate protein toxicity by secreting recombinant proteins into the culture medium. As such, the X-33/pPICZalphaA host-vector system is one of the most widely used platforms for recombinant protein production in *P. pastoris* and has been shown to successfully express many challenging classes of proteins of eukaryotic origin with relatively high yields (Teh et al. 2011; Arjmand et al. 2013; Ünver et al. 2015; Gomez-Ramírez et al. 2023).

In our study, we obtained recombinant production of human ST6GalNAc5 and ST6GalNAc6 in *P. pastoris* and achieved high stability and biological activity. We also investigated the contribution of *N*-glycans to protein activity and stability, highlighting the importance of *N*-glycans in achieving high protein stability and activity in vitro. Additionally, we screened the effects of amino acid supplementation and magnesium addition on the expression and secretion of ST6GalNAc5 and ST6GalNAc6 proteins in the wild-type *P. pastoris* X-33 strain to improve ST6GalNAc5 and ST6GalNAc6 production.

## Materials and methods

### Substrates and analytical standards

Sialyllacto-*N*-tetraose a (LST-a), lacto-*N*-tetraose (LNT), and lacto-*N*-triose II (LNTII) were provided by DSM-Firmenich (Hørsholm, Denmark). Cytidine 5′-monophospho-β-d-N-acetylneuraminic acid sodium salt (CMP-Neu5Ac) and disialyllacto-*N*-tetraose (DSLNT) were purchased from Biosynth (Bratislava, Slovakia). *N*-Acetylneuraminic acid (Neu5Ac) was purchased from Merck (Darmstadt, Germany).

### Synthesis of 3′-sialylgalacto-*N*-biose

The alternative sialyltransferase acceptor substrate 3′-sialylgalacto-*N*-biose (3′-SGNB) was synthesized using the α−2,3-specific *Trypanosoma cruzi* transsialidase (TcTS (Holck et al., 2014)), *N*-acetylneuraminic acid-α2,3-d-galactose (3SGal; Biosynth) as the donor substrate and galacto-*N*-biose (GNB; Biosynth) as the acceptor substrate in a ratio of 3:1 dissolved in PBS buffer (pH 5.9) with a reaction time of 48 h at room temperature. The resulting product and unreacted 3SGal were separated from unreacted GNB and galactose using Dowex88 and Dowex 1X4 resin columns (Merck; elution with water). 3′-SGNB and 3SGal were bound to Dowex 1X4 resin while GNB and Gal eluted. Subsequent separation of 3′-SGNB from unreacted 3SGal was obtained on Dowex 1X4 using bicarbonate gradient elution, and the resulting purity of 3′-SGNB was approx. 90%. The product structure was verified by ^1^H-NMR and ^13^C-NMR on a Bruker Avance 600 MHz and 800 MHz instrument (Bruker Daltonics GmbH, Bremen, Germany), respectively. The chemical shifts matched those previously reported for 3′-SGNB (Li et al., 2015).

### DNA constructs

**Fig. 1:**
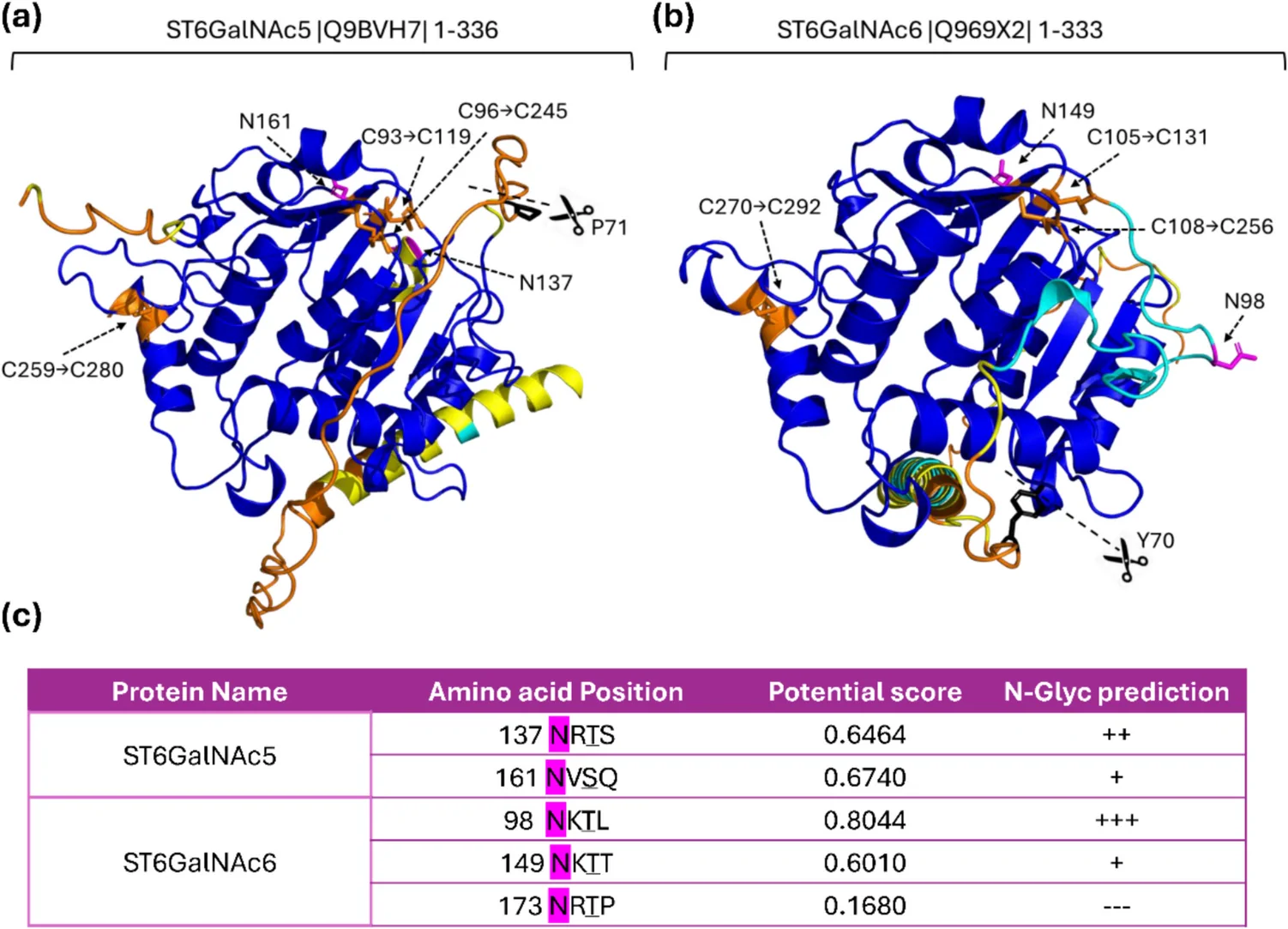
Analysis of the amino acid sequences of human ST6GalNAc5 and ST6GalNAc6 for the design of constructs for expression in yeast. AlphaFold2 models of full-length human **a** ST6GalNAc5 (UniProt Q9BVH7) and **b** ST6GalNAc6 (UniProt Q969X2). Human STs have low sequence similarity but adopt the similar GT-A variant 2 topology, i.e., a seven-stranded β-sheet flanked by multiple helices (Grewal et al., 2021). Conserved cysteines are highlighted in orange, and *N*-glycosylation motifs are highlighted in magenta. Regions with low model confidence scores (yellow, 70 > pLDDT > 50) and very low confidence scores (orange, pLDDT < 50) are indicated. **c** Predicted *N*-glycosylation sites in ST6GalNAc5 and ST6GalNAc6 sequences, as determined by NetNGlyc 1.0. N137 and N161 in ST6GalNAc5 and N98 and N149 in ST6GalNAc6 were predicted to be involved in *N*-linked glycosylation (magenta boxes)

The codon-optimized DNA sequences (Table S1; GenBank accession numbers PV837576 and PV837577), designed according to *Pichia pastoris* codon usage preferences, encoded amino acid residues 72–336 of the human ST6GalNAc5 gene (Uniprot: Q9BVH7) and residues 71–333 of ST6GalNAc6 (Uniprot: Q969X2). These sequences were custom-synthesized and cloned into the pPICZαA integrative vector by GenScript (Piscataway, NJ, USA) employing *Eco*RI and *Sal*I restriction sites (Fig. S1B). The resulting constructs were placed under the control of the methanol-inducible alcohol oxidase (AOX1) promoter and fused to an N-terminal α-mating factor secretion signal leader from *Saccharomyces cerevisiae* via a (GGGS)₂ linker and a His₆ tag (Fig. 1). Propagation of the recombinant vector was carried out using *E. coli* DH5α cells. Successful transformants were selected on low-salt LB agar plates with 25 µg mL^−1^ zeocin and then propagated in low-salt LB medium with 25 µg mL^−1^ zeocin.

### Site-directed mutagenesis on *N*-glycosylation site sequence

Mutations at the glycosylation sites were introduced via point mutagenesis using the pPICZαA-based expression plasmids encoding (GGGS)_2_-His_6_-Δ71ST6GalNAc5 or (GGGS)_2_-His_6_-Δ70ST6GalNAc6 as templates, respectively. First, the N137Q mutation was introduced in Δ71ST6GalNAc5 using Phusion High-Fidelity PCR Master Mix with HF Buffer (Thermo Fisher Scientific, Waltham, MA, USA) and the quick-change primers 1-2 (Table S2). Next, N161Q was introduced using the Δ71ST6GalNAc5 N137Q single-site mutant as a template and the quick-change primers 3-4. The resulting PCR fragments were digested with DpnI (Thermo Fisher Scientific), and the digested product was transformed into competent *E. coli* DH5α cells, and plasmids were extracted using the GeneJET Plasmid Miniprep Kit (Thermo Fisher Scientific). Plasmids were mixed with sequencing primers (5′-TTGCTGCTAAAGAAGAAGGGGTATCTC-3′) and sequenced by Eurofins (Ebersberg, Germany) to confirm the mutation. Introduction of N98Q and N149Q in Δ70ST6GalNAc6 followed a similar protocol using two different pairs of quick-change primers 5-6 and 7-8.

### Transformation and selection of P. pastoris X-33 and KM71H clones

A wild-type *P. pastoris* strain, X-33 (Mut⁺ phenotype), and KM71H (Mutˢ phenotype), transformed with pPICZαA-#1-5, were used in this study. The plasmids were linearized using the FastDigest MssI restriction enzyme (Thermo Fisher Scientific) before transforming electrocompetent *P. pastoris* X-33 and KM71H by electroporation according to Invitrogen (Carlsbad, CA, USA) protocols. Positive transformants were selected on yeast extract peptone dextrose (YPD) agar plates with 100 µg mL^−1^ zeocin. Transformed *P. pastoris* colonies appeared within 72 h at 30 °C. To select high-expressing clones, randomly selected colonies were restreaked onto YPD agar plates with increasing zeocin concentrations (up to 2000 µg mL^−1^). Clones exhibiting hyper-resistance to high zeocin concentrations were selected as an indication of multiple gene insertions in the genome.

### Protein expression

Expression was done according to the Easy-Select™ *P. pastoris* expression kit protocol (Invitrogen, Carlsbad, CA, USA), with incubation at 28 °C and 180 rpm in an orbital shaker for growth on BMGY (1% (w/v) yeast extract, 2% (w/v) peptone, 0.1 M potassium phosphate (pH 6.0), 1.34% (w/v) YNB, 0.0004% (w/v) biotin, and 1% (v/v) glycerol), and zeocin concentration increased to 200 µg mL^−1^ for growth on BMMY (1% (w/v) yeast extract, 2% (w/v) peptone, 0.1 M potassium phosphate (pH 6.0), 1.34% (w/v) YNB, 0.0004% (w/v) biotin, and 0.5% (v/v) methanol). Culture temperature, optical density of cells at the induction point, induction duration, and amount of added methanol during inductions were considered as factors to maximize protein production and secretion*.* Following this optimization, cultures grew under an increasing methanol concentration: 0–24-h at 28 °C with 0.5% methanol; 24–48-h at 20 °C with 1% methanol; 48–144-h at 20 °C with 2% methanol. These conditions were established by adding absolute methanol at a final concentration of 0.5–2.0% (v/v) every 24 h to maintain induction. Shaking at 130 rpm was used for protein expression cultivation. Protein production in the *Pichia* X-33 clones was increased by supplementing the cultures with 0.5–3.0 mM MgCl₂ and/or 0.5–2.0% OmniPur® Casamino acids (Sigma-Aldrich, Steinheim, Germany) at 0 and/or 72 h post-induction. Three supplementation regimes for Casamino acids were tested: (1) 0.5%, 1.0%, or 2.0% at 72 h post-induction; (2) 1.0% at both 0 and 72 h post-induction.

### Analysis of protein expression and secretion during fermentation

To analyze the protein content in daily samples from *Pichia pastoris* cultures, 4-mL aliquots of the cell culture were collected, centrifuged for 10 min at 10,000*g* and 4 °C, and the resulting supernatants and cell pellets were retained. Cell pellets were resuspended in 500 µL of 50 mM HEPES, 100 mM NaCl, lysed using CelLytic™ Y Cell Lysis Reagent (Sigma-Aldrich, Steinheim, Germany), and analyzed by Western blot using an anti-His antibody as described below. Supernatants were concentrated 20 times using Vivaspin® 500, 10 kDa MWCO polyethersulfone spin filters (Sigma-Aldrich, Steinheim, Germany), and 20 µL of each sample was used in the sialylation assay, as described below.

### Protein purification

The purification of recombinant proteins was performed from cell-free supernatants. After centrifugation (5000*g*, 30 min, 4 °C) and filtering (0.45 µm), the supernatant was concentrated using a 10-kDa PES crossflow membrane (Vivaflow® 200; Sartorius, Göttingen, Germany) with buffer exchange in 50 mM Trizma (pH 7.0) and 200 mM NaCl. The concentrated supernatant was supplemented with 25 mM imidazole (pH 7.0) and applied to a 2-mL pre-equilibrated HisPur™ Ni-NTA Resin (Thermo Fisher Scientific) for binding. After binding, the resin was washed twice with 10 column volumes (CV) of washing buffer (50 mM Trizma, pH 7.0, 200 mM NaCl, 25 mM imidazole) and eluted stepwise with 50 mM Trizma, pH 7.0, 200 mM NaCl, and 400 mM imidazole. Collected fractions were desalted using Sephadex G-25 PD-10 columns (Cytiva, Uppsala, Sweden) with 50 mM HEPES (pH 7.0) and 100 mM NaCl as the desalting buffer. Following concentration on a 10-kDa PES membrane (Vivaspin 20; Sartorius), protein concentration was determined by measuring absorption at 280 nm and using the calculated extinction coefficients 40,910 M^−1^ cm^−1^ for ST6GalNAc5 and 50,880 M^−1^ cm^−1^ for ST6GalNAc6. For Strep II tagged proteins, purification was performed using IBA Lifesciences Strep-Tactin™XT 4Flow™ Resin (Fisher Scientific, Gothenburg, Sweden) according to the manufacturer’s recommendations. Proteins were visualized on SDS-PAGE (Biorad, Hercules, CA, USA). His-tagged and Strep-tagged proteins were detected by Western blot using mouse monoclonal peroxidase-conjugated Anti‐His-tag (Sigma-Aldrich, Steinheim, Germany) or Anti‐Strep-tag-II (IBA Lifesciences, Göttingen, Germany) antibodies as described previously (Madsen et al., 2024).

### Protein deglycosylation

To test the effect of enzymatic protein deglycosylation, purified proteins were treated with Endo H or PNGase F (New England Biolabs, Ipswich, MA, USA) according to the manufacturer’s instructions. In short, 5–15 µg of desalted protein was digested with 500 units of PNGase F or Endo H at 37 °C for 2–20 h in a non-denaturing buffer. For the control, the protocol was performed with all components included, except for PNGase F and Endo H.

### Activity tests and kinetics measurements

Reactions were conducted using 1 mM CMP-Neu5Ac and 1 mM LST-a and 1–20 µM protein in either 50 mM MES pH 5.5, 50 mM MES pH 6.0, 50 mM MES pH 6.5, or 50 mM HEPES pH 7.0 buffers. Reactions took place at 37 °C and 450 rpm for 1–20 h. Reactions were stopped by 10 min incubation at 95 °C.

For kinetic measurements, the 200 µL assay contained 50 mM HEPES (pH 7.0), 1.5 µM purified ST6GalNAc5 or 2.0 µM ST6GalNAc6, a fixed concentration of CMP-Neu5Ac (5 mM), and varying concentrations of LST-a (0.2–10 mM). Reactions were incubated at 37 °C and 450 rpm for 180 min (ST6GalNAc5) or 60 min (ST6GalNAc6). Each reaction was stopped by a 10-min incubation at 95 °C and the amount of DSLNT, CMP-Neu5Ac, Neu5Ac, and LST-a was quantified by HPLC (below). Kinetic parameters (*K*_m_ and *V*_max_) were determined by fitting experimental data to the Michaelis-Menten equation, using SigmaPlot v.16 software package (Systat Software, Inc.).

### Carbohydrate analysis

Reactions were followed by thin-layer chromatography (TLC) by applying 1.5-µL reaction mixtures on silica gel 60 TLC aluminum sheets (Merck) together with relevant standards. The mobile phase was BuOH/AcOH/H_2_O (1.4:1:1; all solvents from Merck). Spots on the plate were developed by 10% sulfuric acid in ethanol and subsequent heating.

Formation of DSLNT was quantified by HPLC on a Thermo Scientific UltiMate 3000 HPLC with charged aerosol detection (CAD) using a Thermo Scientific Acclaim Trinity P2 column (100 × 2.1 mm, 3 µm) at 30 °C. The flow rate was 0.4 mL min^−1^ and the eluent system comprised acetonitrile (A), MilliQ water (B), and 100 mM ammonium formate in MilliQ water, pH adjusted to 3.65 with formic acid (C). Elution was performed with a linear gradient starting at 72% A, 24% B, and 4% C, then changed to 44% A and 56% C over the first 10 min, then to 40% A and 60% C over the next 2 min, and a subsequent isocratic step at the same composition for 3 min. The column was then re-equilibrated at 72% A, 24% B, and 4% C for 5 min. External calibration was used for LST-a and Neu5Ac with five points in a concentration range of approx. 0.02–0.3 g L^−1^. CMP, CMP-Neu5Ac, and DSLNT were identified by retention time comparison with reference materials. CMP was quantified as Neu5Ac equivalent, while CMP-Neu5Ac and DSLNT as LST-a equivalents.

The presence of DSLNT was confirmed by LC-MS on a Thermo Scientific UltiMate 3000 HPLC coupled to a Bruker microTOF quadrupole-time-of-flight mass spectrometer, using a Thermo Scientific Accucore Amide-HILIC column (150 × 3 mm, 2.6 µm) at 25 °C. The flow rate was 0.8 mL min^−1^ and the eluent system comprised acetonitrile (A) and 20 mM ammonium formate in MilliQ water, pH adjusted to 3.0 with formic acid (C). Elution was performed with a linear gradient starting at 72% A and 28% B, then changed to 68% A and 32% B over the first 5 min, then to 60% A and 40% B over the next 3 min, and a subsequent isocratic step at the same composition for 2 min. The column was then re-equilibrated at 72% A and 28% B for 3 min. The mass spectrometer was used in negative mode and full scan. DSLNT was confirmed by both retention time comparison with the DSLNT standard and by the main spectral line at 643.7, corresponding to the doubly charged molecular ion (M-2H)^2−^.

To separate DSLNT from any isomers (Pei et al. 2024), we employed high-performance anion-exchange chromatography with pulsed amperometric detection (HPEAC-PAD) using a Thermo Scientific ICS-5000 and a Thermo Scientific CarboPac PA210 (150 × 4 mm, 4 µm) column at 15 °C. The flow rate was 0.5 mL min^−1^ and the eluent system comprised MilliQ water (A), 100 mM sodium hydroxide in water (B), 125 mM sodium acetate and 100 mM sodium hydroxide in water (C), and 500 mM sodium hydroxide in water (D). Elution was performed with a linear gradient starting at 85% A, 10% B, and 5% C, then changed to 80% A, 10% B, and 10% C over the first 36 min, then to 10% B and 90% C over the next 10 min, and a subsequent isocratic step at the same composition for 10 min. The column was then regenerated at 100% D for 10 min, and re-equilibrated at 85% A, 10% B, and 5% C for 20 min. DSLNT was identified by retention time comparison with the external standard. The structural isomer was prepared as a transsialylation reaction between 6’SL (Biosynth) and LST-a catalyzed by a bacterial GT80 α2,6-sialyltransferase known to sialylate O-6 of the terminal and internal galactose units (Yu et al., 2014); no structural determination was performed for this isomer, but it could be separated from DSLNT by HPAEC-PAD.

### NanoDSF analysis

Thermal unfolding measurements were performed via NanoDSF analysis using a Prometheus Panta instrument (NanoTemper Technologies, Munich, Germany) as described previously (Vuillemin et al., 2024). Purified protein samples (5 µM) were prepared in 50 mM HEPES (pH 7.0) or 50 mM Tris (pH 7.0) with 100 mM NaCl.

### Circular dichroism measurements

Circular dichroism (CD) spectroscopy analysis of purified wild-type and mutant ST6GalNAc5 and ST6GalNAc6 was performed in 20 mM potassium phosphate buffer (pH 7.0), with and without 10 mM NaCl, at a protein concentration of 0.4 mg/mL. Measurements were carried out using a JASCO J-1500 spectrophotometer and a 1 mm path length quartz cuvette (Hellma® absorption cuvette, Sigma-Aldrich, Steinheim, Germany). Spectra were recorded over a wavelength range of 190–250 nm at 15 °C with a 1.0-nm bandwidth and a scanning speed of 100 nm/min. For each sample, the average of five spectra was collected. The secondary structure of each sample was analyzed using CDPro software (Sreeram and Woody 2000).

### Statistical analysis

Unless otherwise indicated, all data points represent average values from at least three independent experiments. For comparisons involving multiple groups, Tukey’s HSD test was used to assess differences between groups using JMP Pro 16.0.0 (SAS Institute, Cary, NC, USA).

## Results

### Design of expression constructs

To optimize ST6GalNAc5 and ST6GalNAc6 for successful expression in yeast, we analyzed their protein sequences for disordered regions and post-translational modification sites. Within the ST6GalNAc subfamily, only the structure of ST6GalNAc2 has been determined, revealing the sialylmotif scaffold that forms the CMP-Neu5Ac binding site (Moremen et al., 2018). ST6GalNAc2 shares only 23.6% and 24.5% protein sequence identity with ST6GalNAc5 and ST6GalNAc6, respectively. Both ST6GalNAc5 and ST6GalNAc6 are predicted to have a short cytoplasmic tail at the N-terminus, followed by a single transmembrane domain, a non-conserved linker (referred to as the stem region), and the C-terminal catalytic domain oriented towards the luminal side (Figure S1c). ST6GalNAc5 and ST6GalNAc6 both contain three pairs of cysteines in the C-terminal domain (Fig. 1a, b and FigureS1), which are predicted to be involved in proper protein folding, thermal stability, and enzymatic activity due to their contribution to conserved intrachain disulfide bonds within the sialylmotifs, which are essential for binding both donor and acceptor substrates (Datta et al. 2001; Ortiz-Soto et al., 2019).

**Fig. 2:**
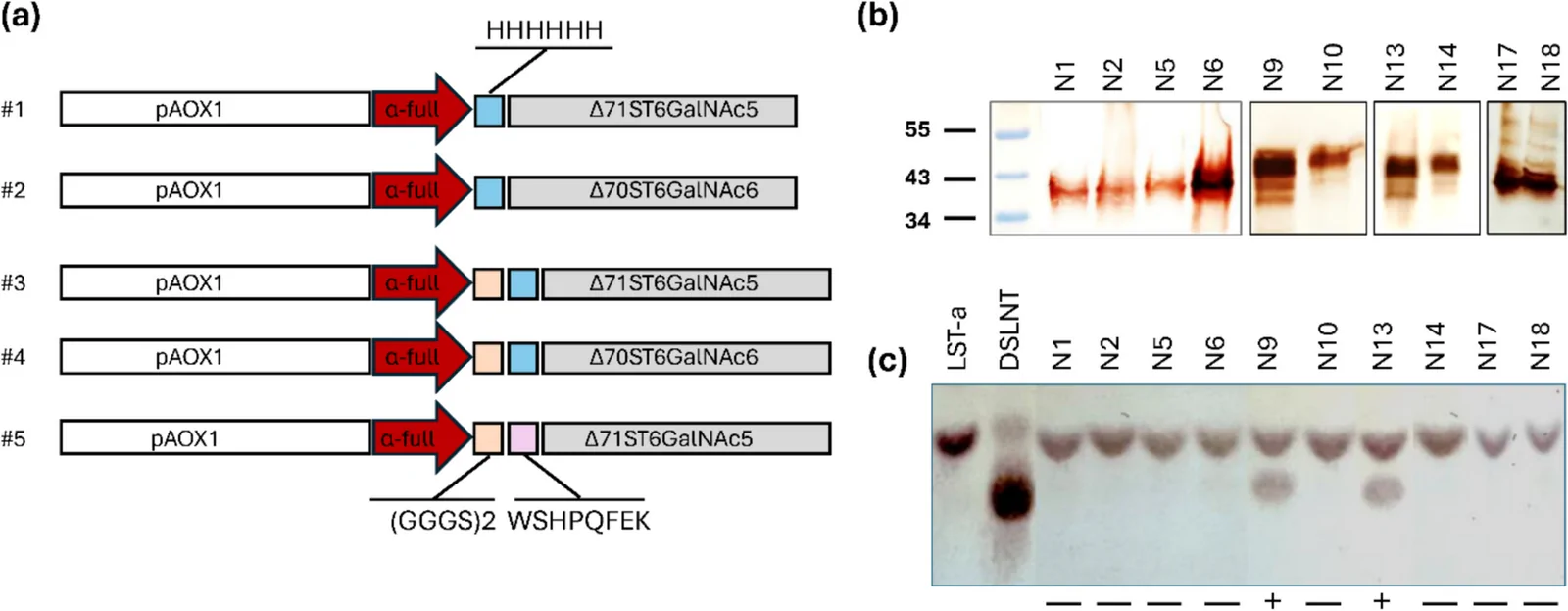
Effect of Mut phenotype and construct design on Δ71ST6GalNAc5 andΔ70ST6GalNAc6 expression in *Pichia pastoris* at 20 and 28 °C. **a** Schematic illustration of gene constructs designed for *P. pastoris* expression based on the pPICZαA vector. **b** Detection of target proteins in concentrated *P. pastoris* cell culture supernatants detected by Western blotting, where N1-N18 corresponds to the combinations of constructs and expression conditions given in Table 1. **c** A representative silica gel thin-layer chromatography (TLC) plate shows the LST-a acceptor substrate and the DSLNT product resulting from α−2,6-sialylation catalyzed by purified protein samples expressed in *P. pastoris* at 20 °C. In each reaction, 3 µM of purified protein was incubated overnight at 37 °C with 1 mM LST-a and 1 mM CMP-Neu5Ac in a buffer containing 50 mM HEPES (pH 7.0) and 100 mM NaCl. Compounds were separated by TLC using butanol-acetic acid-water (1.4:1:1, v/v/v) as the mobile phase. Uncropped versions of Western blots are available in Figure S8

We analyzed the AlphaFold2-generated models of ST6GalNAc5 and ST6GalNAc6, identifying predicted unstructured and low-confidence regions (pLDDT < 70) (Fig. 1a, b). These regions, often indicative of protein disorder (Guo et al., 2022), are located within the highly variable stem region of STs. Based on these findings, we designed constructs encoding amino acids 72–336 of ST6GalNAc5 and 71–333 of ST6GalNAc6. A His_6_ tag (four constructs, #1-4) or a Strep II tag (one construct, #5) was added to the N-terminus of the proteins (Fig. 2a). To achieve secretion in yeast, the genes of interest were fused at the N-terminus to an α secretion factor (Brake et al., 1984), which is known to direct the secretion of reporter proteins into the culture medium. In three constructs (#3-5), a (GGGS)₂ linker peptide was included (Fig. 2a).

ST6GalNAc5 and ST6GalNAc6 each contain two *N*-linked glycosylation sites as predicted by the NetNGlyc 1.0 server (Blom et al., 2004): sites with a glycosylation potential ≥ 0.5 were considered likely to be glycosylated in vivo (Fig. 1c). In ST6GalNAc5, the predicted *N*-glycosylation sites are located at N137 and N161, proximal to the sialylmotif L (amino acids 93–136). Similarly, the *N*-linked glycosylation sites in ST6GalNAc6 are at N98 and N149, near sialylmotif L (amino acids 105–148) (FigureS1).

### Comparison of Mut⁺ and Mutˢ strains for recombinant protein production

Construct design, different phenotypes, and incubation temperature can profoundly influence protein stability, solubility, and expression levels in yeast. Therefore, we compared the effects of protein expression at either 20 °C or 28 °C in Mut⁺ and Mutˢ backgrounds by using either X-33 or KM71H *P. pastoris* strains modified with pPICZαA. During the production phase, when methanol was used as the sole carbon source, both strains displayed identical cell growth during the first 24 h at 20 °C. However, thereafter the KM71H strain grew slower, reflecting its slower methanol consumption phenotype, consistent with previous reports (Krainer et al., 2012) (Figure S2a). In cultures grown at 28 °C, significant cell proteolysis was observed starting 72 h post-induction.

Recombinant Δ71ST6GalNAc5 and Δ70ST6GalNAc6 were recovered from the culture supernatants as well as from cell lysates using affinity purification to quantify both purified protein yields and the percentage of secreted protein. Attempts to recover recombinant constructs #1-2 without the (GGGS)_2_ linker from *P. pastoris* X-33 and KM71H supernatants were unsuccessful. Very little protein was secreted (< 300 µg/L of cell culture) regardless of the strain or temperature used (Table 1). The majority of the produced protein appeared to remain intracellular. In contrast, proteins from X-33/#3-5 and KM71H/#5 were effectively secreted and accumulated in the culture medium after low-temperature secretory expression in shake flasks, although the amount of secreted protein varied (Table 1). The highest total protein expressions were observed with KM71H/#5, X-33/#5, and X-33/#3 clones. The total protein yields for these transformants ranged from 5.7 to 6.8 mg/L of culture, achieving a secretion efficiency of 45–50%. Analysis of cell and supernatant fractions across different time points (24–144 h) revealed that the first protein signals appeared in the cells 24 h after induction. After each subsequent methanol addition, a portion of the protein was consistently retained in the cells (Figure S3a). Secreted protein was detected in the culture supernatant from 72 h post-induction, with the highest yield observed 144 h after induction (Figure S3a). Monitoring yields of secreted protein and its activity up to 168 h, it was evident that 144 h could be selected as the optimal harvest time as neither secretion nor activity increased significantly beyond 144 h (Figure S3b-c). The Western blot analysis of the concentrated cell culture supernatants (specific antibodies against the His_6_ tag (#1-4) or StrepII tag (#5); Fig. 2b) revealed the presence of the proteins with molecular masses close to or slightly larger than the predicted ones (~ 32 kDa). An attempt to improve the expression of the two best-expressing constructs X-33/#3, X-33/#5, and KM71H/#5 by co-feeding with 0.1% (v/v) glycerol resulted in significant decreases in protein yields and secretion efficiency (Table S3).

**Table 1:** Analysis of protein expression after 144 h of fermentation at either 20 °C or 28 °C. The secretion-to-total protein ratio was determined for the five constructs expressed in the X-33 and KM71H strains at 20 °C and 28 °C, unless cell proteolysis was observed. The constructs contain either a direct fusion (#1 and #2 for His₆-tagged Δ71ST6GalNAc5 and Δ70ST6GalNAc6) or a fusion via a linker (#3 and #4 for His₆-tagged Δ71ST6GalNAc5 and Δ70ST6GalNAc6, and #5 for Strep-tagged Δ71ST6GalNAc5) to the α-secretion factor. Construct #5 was not tested at 28 °C based on the issues with cell proteolysis and low secretion observed at this temperature for the other Δ71ST6GalNAc5 constructs (#1 and #3). “n/d” in the activity column indicates that activity was not detected, whereas “-” indicates that no analysis was made (or cell proteolysis was not observed). Values are given as mean ± standard deviation (*n* = 2); superscript letters a–e indicate significant differences between values (*p* < 0.05)

#	Host/construct	T (°C)	Cell proteolysis	Activity	Secreted (mg/L)	Total protein (mg/L)	Secretion (%)
N1	X-33/#1	20	-	n/d	0.12 ± 0.02^e^	4.50 ± 0.40^bcd^	2.55 ± 0.11^d^
N2	KM71H/#1	20	-	n/d	0.10 ± 0.01^e^	2.90 ± 0.20^cde^	3.30 ± 0.40^d^
N3	X-33/#1	28	48 h	-	-	-	-
N4	KM71H/#1	28	72 h	-	-	-	-
N5	X-33/#2	20	-	n/d	0.12 ± 0.02^e^	4.85 ± 0.25^abc^	1.90 ± 0.00^d^
N6	KM71H/#2	20	-	n/d	0.13 ± 0.03^e^	2.55 ± 0.15^de^	4.98 ± 1.27^ cd^
N7	X-33/#2	28	-	n/d	0.12 ± 0.02^e^	2.30 ± 0.20^e^	4.98 ± 0.22^ cd^
N8	KM71H/#2	28	-	n/d	0.33 ± 0.03^de^	4.60 ± 0.40^abc^	6.97 ± 0.18^ cd^
N9	X-33/#3	20	-	Active	2.70 ± 0.20^b^	5.70 ± 0.20^ab^	47.33 ± 1.83^a^
N10	KM71H/#3	20	-	n/d	0.48 ± 0.08^de^	5.80 ± 0.30^ab^	8.14 ± 0.87^ cd^
N11	X-33/#3	28	72 h	-	-	-	-
N12	KM71H/#3	28	-	n/d	0.78 ± 0.08^d^	4.35 ± 0.45^bcde^	17.83 ± 0.12^b^
N13	X-33/#4	20	-	Active	1.90 ± 0.20^c^	4.20 ± 0.20^bcde^	43.80 ± 1.30^a^
N14	KM71H/#4	20	-	n/d	0.63 ± 0.13^de^	5.15 ± 0.85^ab^	12.06 ± 0.44^bc^
N15	X-33/#4	28	72 h	-	-	-	-
N16	KM71H/#4	28	72 h	-	-	-	-
N17	X-33/#5	20	-	n/d	2.95 ± 0.15^ab^	5.85 ± 0.15^ab^	50.53 ± 3.86^a^
N18	KM71H/#5	20	-	n/d	3.33 ± 0.13^a^	6.75 ± 0.35^a^	49.30 ± 0.70^a^

Subsequently, both the culture supernatants from each expression condition, along with fractions of purified proteins, were assessed by TLC for their ability to form DSLNT from CMP-Neu5Ac and LST-a (Fig. 2c). Functional expression was achieved only for X-33/#3 and X-33/#4, i.e., His_6_-Δ71ST6GalNAc5 and His_6_-Δ70ST6GalNAc6 from the (GGGS)_2_ linker constructs, induced at 20 °C with 0.5–2.0% methanol and a post-induction time of 144 h. Thus, despite the high protein yields obtained with the Strep II-tag constructs KM71H/#5 and X-33/#5, no activity was detected in the purified proteins. We assume that the buffer system optimal for the binding and elution of Strep II–tagged ST6GalNAc5 (100 mM Tris (pH 8.0), 150 mM NaCl, 1 mM EDTA, and 50 mM biotin for elution) was not suitable for maintaining the stability and activity of ST6GalNAc5.

### Magnesium and casamino acid supplementation enhances recombinant protein production

Our preliminary results demonstrated a relatively low expression response to increasing doses of methanol, with a maximum of 3.3 mg of secreted protein per liter of culture (Table 1). To assess the effects of supplements on ST6GalNAc5 (X-33/#3) and ST6GalNAc6 (X33/#4) expression, cells were cultured in BMMY medium supplemented with varying amounts of MgCl_2_ and Casamino acids (CA).

**Fig. 3:**
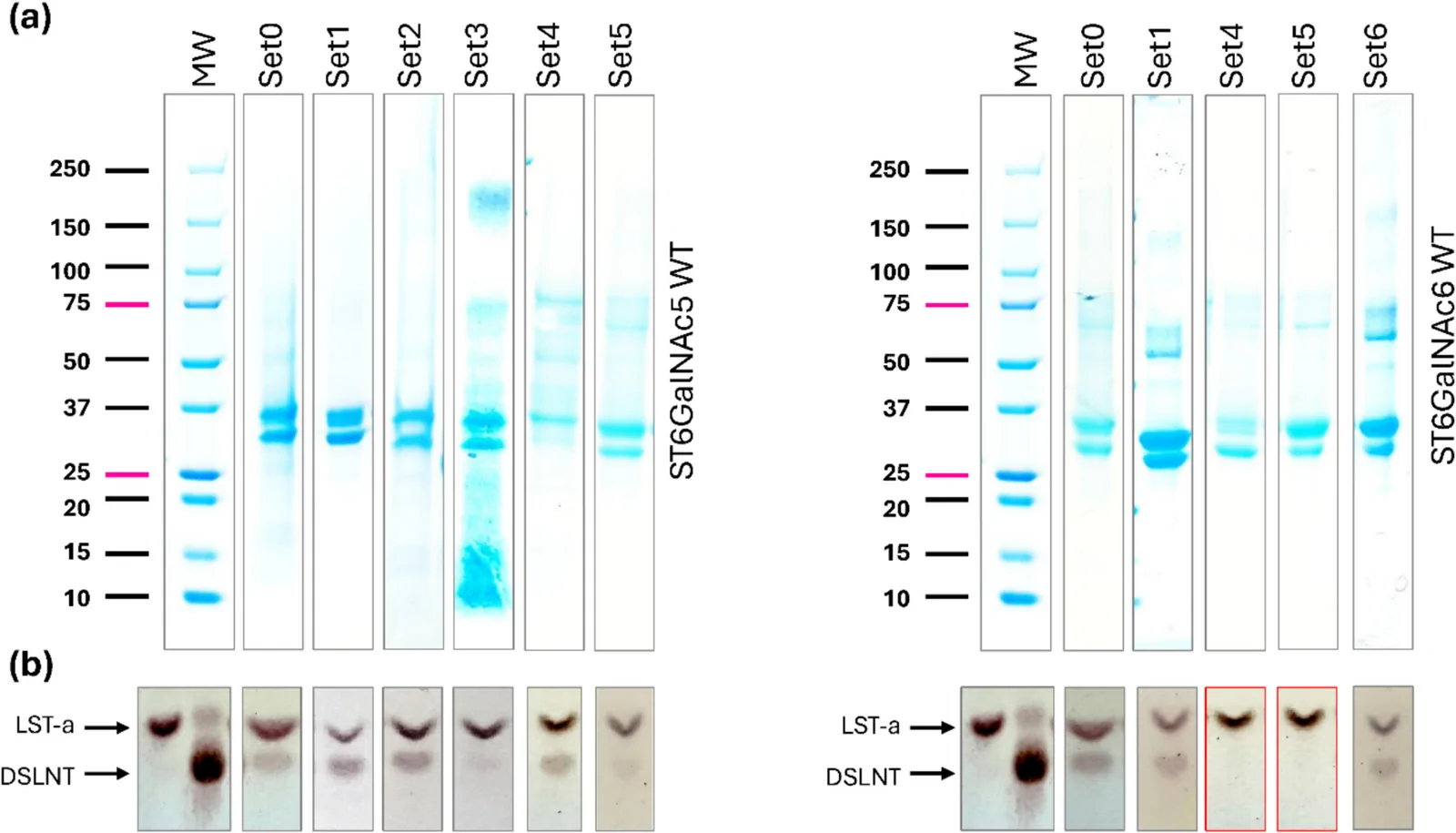
Protein quality when using Casamino acids and magnesium supplements in *Pichia pastoris* X-33 shake flask experiments. **a** SDS-PAGE analysis of purified proteins (~ 20 µg). The apparent molecular weights (MW) of the purified samples are consistent. **b** TLC plate panel showing the reaction product (DSLNT) obtained from the sialylation reaction with LST-a (acceptor) and CMP-Neu5Ac (donor) using ~ 5 µM purified protein. Reactions where no DSLNT was detected are highlighted with red boxes. Uncropped versions of SDS-PAGE gels are available in FigureS9. Supplementation and growth conditions were as follows (Table 2): Set 0 (no additions); Set 1 (3 mM MgCl₂ added after 72 h); Set 2 (3 mM MgCl₂ and 2% CA added after 72 h); Set 3 (3 mM MgCl₂ and 2% CA added after 72 h, growth at 28 °C); Set 4 (3 mM MgCl₂ and 1% CA added after 72 h); Set 5 (3 mM MgCl₂ and 1% CA added twice after 0 and 72 h, respectively); and Set 6 (0.5 mM MgCl₂ and 0.5% CA added after 72 h). Unless otherwise stated, cultures were grown at 20 °C.

With continuous methanol induction at 20 °C, *P. pastoris* X-33 maintained continuous growth for 144 h, including a 48-h stationary phase, reaching a final OD_600_ of approximately 25 without any additional supplementation. The addition of magnesium and CA at the end of the exponential growth phase further boosted cell growth, resulting in higher final cell densities of around 40 (Figure S2b). The addition of MgCl_2_ (0.5–3.0 mM) and CA (0.5–2.0%) also enhanced the recombinant production of ST6GalNAc5 and ST6GalNAc6. The highest expression was achieved with two rounds of 1.0% CA supplementation (Fig. 3a; Table 2). Notably, even MgCl_2_ supplementation alone at 72 h post-induction altered both total and secreted expression of ST6GalNAc5 and ST6GalNAc6. In the case of ST6GalNAc5, adding only magnesium to the induction media increased secreted expression 1.2-fold to 3.3 g/L of protein (Table 2). In the case of ST6GalNAc6, a 3.4-fold increase in expression was observed with a lower concentration of MgCl₂, specifically 0.5 mM.

**Table 2:** Analysis of protein expression using the X-33/#3 (Δ71ST6GalNAc5) and X-33/#4 constructs (Δ70ST6GalNAc6) and supplementation with MgCl_2_ and Casamino acids at specific times post-induction at varying expression temperatures (20 °C or 28 °C). For Set5, MgCl_2_ and Casamino acids were added twice during growth (after 0 and 72 h post-induction, respectively). Values are given as mean ± standard deviation (*n* = 2); superscript letters a–e indicate significant differences between values (*p* < 0.05). “n/d” in the activity row indicates that activity was not detected, whereas “+” indicates that activity was detected but low, “+++ ” indicates the highest level of activity observed in the study (by TLC), and “-” indicates that no analysis was made

Conditions	Set0	Set1	Set2	Set3	Set4	Set5		Set6
*T* (°C)	20	20	20	28	20	20		20
Suppl. time (h)	-	72	72	72	72	0	72	72
MgCl_2_ (mM)	0	3	3	3	3	3	3	0.5
Casamino acids (%)	0	0	2	2	1	1	1	0.5
**ST6GalNAc5 WT**								
Secreted (mg/L)	2.7 ± 0.2^e^	3.3 ± 0.2^e^	6.5 ± 0.3^ cd^	5.3 ± 0.2^d^	8.0 ± 0.5^bc^	10.3 ± 0.7^a^		-
Total protein (mg/L)	5.7 ± 0.2^d^	9.2 ± 0.6^ cd^	14.0 ± 1.0^bc^	10.0 ± 0.6^ cd^	16.5 ± 0.6^bc^	29.0 ± 4.0^a^		-
Secretion (%)	47 ± 1.8^abc^	36 ± 0.8^c^	46 ± 5.2^abc^	53 ± 1.3^ab^	49 ± 1.3^abc^	36 ± 2.5^c^		-
Activity (TLC)	+++	+++	+++	+	+++	+		-
**ST6GalNAc6 WT**								
Secreted (mg/L)	1.9 ± 0.2^e^	1.5 ± 0.1^e^	-	-	5.4 ± 0.4^d^	9.2 ± 0.5^ab^		6.5 ± 0.2^ cd^
Total protein (mg/L)	4.2 ± 0.2^d^	3.6 ± 0.4^d^	-	-	9.3 ± 0.4^ cd^	19.1 ± 2.0^b^		10.9 ± 0.2^ cd^
Secretion (%)	44 ± 1.3^bc^	41 ± 3.3^bc^	-	-	58 ± 1.7^a^	48 ± 2.4^abc^		60 ± 1.2^a^
Activity (TLC)	+++	+++	-	-	n/d	n/d		+++

The addition of CA along with magnesium enhanced secreted expression of ST6GalNAc5 by 2.4 to 3.8-fold, with the highest level of 10.3 mg/L achieved through two rounds of supplementation (Table 2). At the same conditions, the expression of ST6GalNAc6 was enhanced to a maximum 4.8-fold increase (9.2 mg/L) compared to control conditions. The increased levels of secreted expression were proportional to the increase in total protein expression. However, no significant increase in the secreted-to-total protein ratio was observed.

Successful purification of ST6GalNAc5 and ST6GalNAc6 was confirmed by SDS-PAGE (Fig. 3a). Along with improved protein expression levels, we observed that magnesium supplementation affected the activity state of recombinantly produced ST6GalNAc6 (to a greater extent) and ST6GalNAc5 (to a lesser extent). The activities of ST6GalNAc5 produced at 20 °C with the addition of MgCl_2_ and CA were sustained in most tests (Table 2 and Fig. 3b), although higher temperature (28 °C) and increasing the MgCl_2_ level by adding 3 mM twice both decreased enzyme activity. In a set of experiments where CA (1.0%) and MgCl_2_ (3.0 mM) were added either individually or together at 0 and 72 h post-induction (Set 4 and Set 5), ST6GalNAc6 showed no signs of activity. ST6GalNAc6 appeared more sensitive to additions of both supplements and produced the highest yield of active, secreted protein (6.5 mg/L) at 0.5 mM MgCl_2_ and 0.5% CA (Table 2 and Fig. 3b).

### Characterization of purified recombinant Δ71ST6GalNac5 and Δ70ST6GalNAc6

**Fig. 4:**
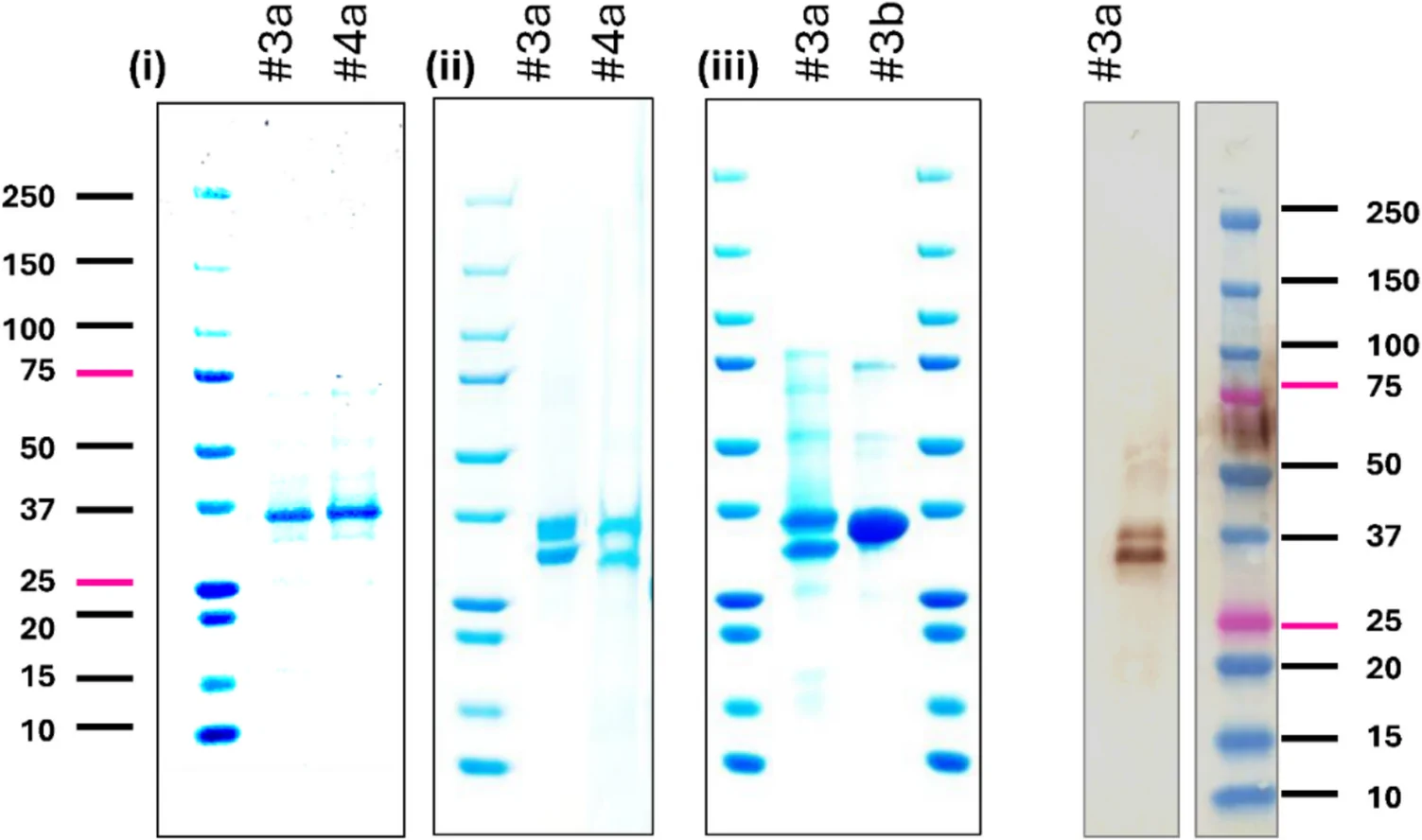
Characterization of purified recombinant 71ST6GalNac5 and Δ70ST6GalNAc6. Left: SDS-PAGE gel of IMAC-purified, secreted Δ71ST6GalNAc5 (#3) and Δ70ST6GalNAc6 (#4) from *Pichia pastoris* cell culture supernatant, where “a” indicates purification from the cell culture supernatant at different protein loads, (i) 8 µg and (ii) 20 µg, and (iii) compares the protein (20 µg) purified from the supernatant to Δ71ST6GalNAc5 purified from the lysed cells (b). Right: Western blot of purified Δ71ST6GalNAc5 (X-33/#3) probed with monoclonal anti-His antibody; an uncropped version is available in Figure S6

After IMAC purification, ST6GalNAc5 (X-33/#3) and ST6GalNAc6 (X-33/#4) exhibited high purity on SDS-PAGE, making additional purification steps unnecessary. Target proteins were the most abundant products, accounting for more than 95% of the total purified protein (Fig. 4). As predicted from the amino acid sequences, the molecular masses of Δ71ST6GalNAc5 (#3) and Δ70ST6GalNAc6 (#4) were ~32.1 kDa and ~31.7 kDa, respectively. However, the apparent molecular weights of the target bands were slightly larger than their theoretical values, likely due to *N*-glycosylation at the predicted sites (Fig. 1c) as *P. pastoris* possesses a lower eukaryotic-type post-translational modification system. With higher protein loads (20 µg) on the gel, the enzymes appeared as double bands on SDS-PAGE and Western blot (Fig. 4). In contrast, Δ71ST6GalNAc5 from lysed cells appeared as a single protein band on SDS-PAGE, suggesting incomplete processing of the protein (Fig. 4).

**Fig. 5:**
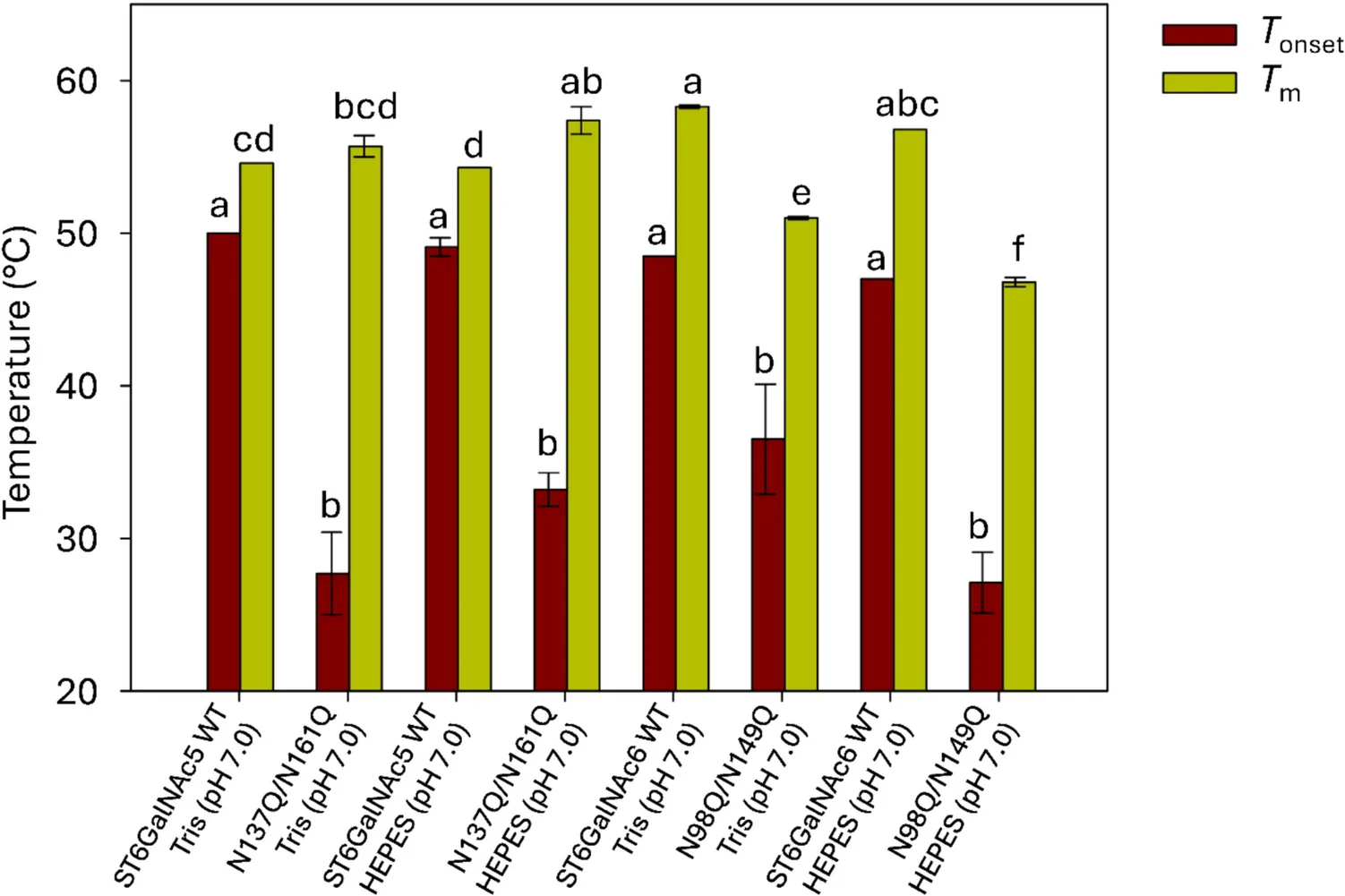
Thermostability values derived from NanoDSF analysis of wild-type (WT) and *N*-glycosylation mutant proteins in 50 mM Tris and HEPES buffers. Values are given as mean ± standard deviation (*n* = 2) and superscript letters a–e indicate significant differences between values (*p* < 0.05)

To assess the thermal stability of the recombinant proteins, freshly purified Δ71ST6GalNAc5 and Δ70ST6GalNAc6 were subjected to label-free NanoDSF analysis. Since the physiological pH of most human tissues is around 7.0–7.4, with some exceptions (Gaohua et al., 2021), protein stability was measured in buffer containing 50 mM HEPES (pH 7.0) and 100 mM NaCl. The onset-of-melting temperatures were 47–49 °C, while the midpoint melting temperatures (*T*_m_) were 54.3 °C for Δ71ST6GalNAc5 and 56.8 °C for Δ70ST6GalNAc6 (Fig. 5).

**Fig. 6:**
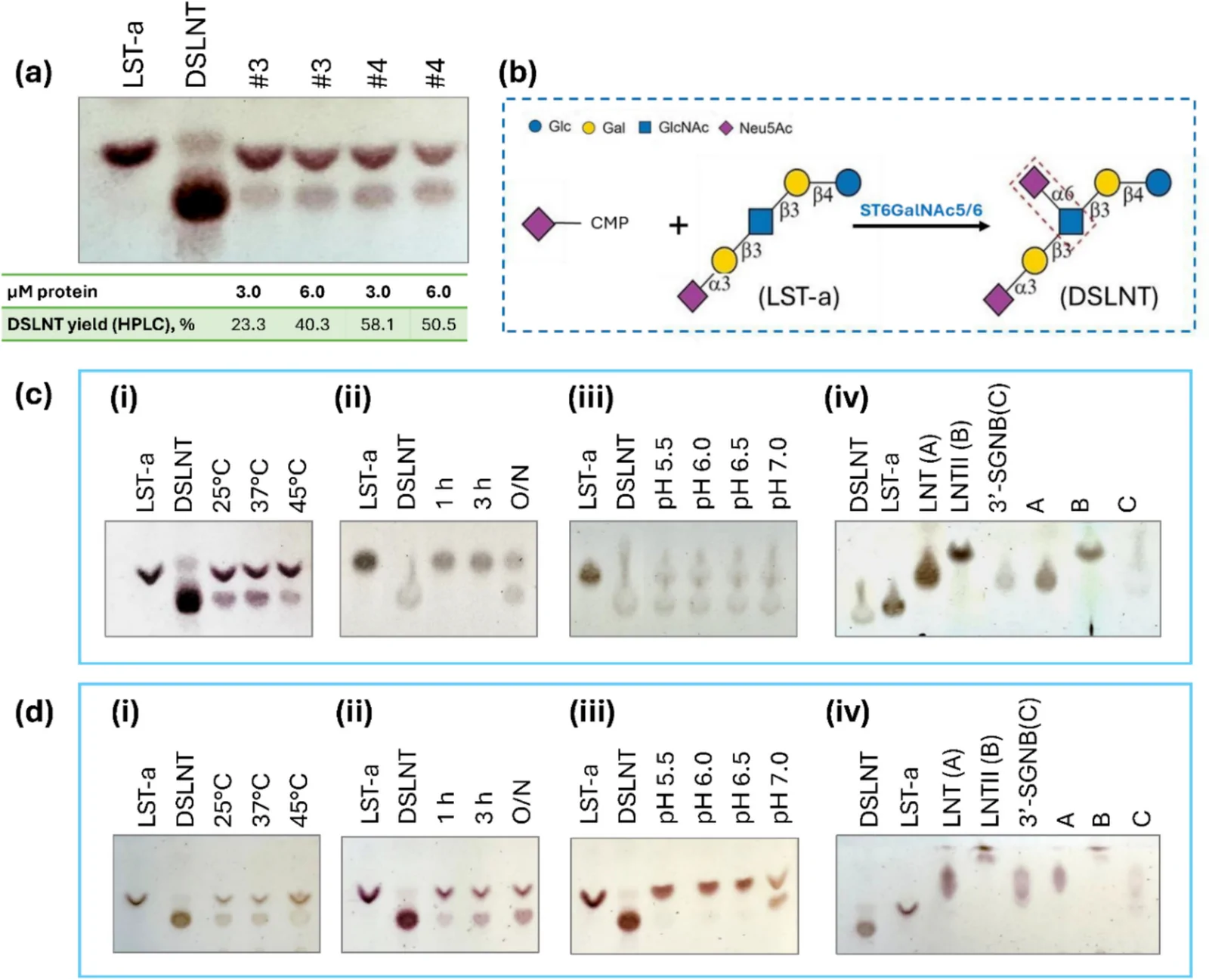
TLC and HPLC analysis of sialyltransferase activity of recombinant Δ71ST6GalNAc5 and Δ70ST6GalNAc6. (**a**) TLC and HPAEC quantitation of DSLNT (formed product) from the reaction with 1 mM CMP-Neu5Ac (donor) and 1 mM LST-a (acceptor) assessed at different concentrations of Δ71ST6GalNAc5 (#3) and Δ70ST6GalNAc6 (#4), measured at 37 °C, pH 7.0, after overnight (O/N) incubation unless otherwise stated. (**b**) Schematic illustration of the reaction leading to DSLNT formation. In the DSLNT structure, the α−2,6-linkage formed between sialic acid and GlcNAc catalyzed by ST6GalNAc5 and ST6GalNAc6 is highlighted. (**c**) Formation of DSLNT with 2.5 µM Δ71ST6GalNAc5 and (**d**) with 2.5 µM Δ71ST6GalNAc6 was monitored at (i) different temperatures, (ii) different time points, (iii) different pH conditions, and (iv) in the presence of three alternative acceptors LNT (A), LNTII (B), and 3′-SGNB (C)

To ascertain that the function of *P. pastoris*–produced proteins was correctly assigned and that the investigated proteins were not only folded in a stable conformation, but also active, purified proteins were further screened for their natural activities. The recombinant Δ71ST6GalNAc5 and Δ70ST6GalNAc6 were confirmed to be active by assessing their ability to sialylate the LST-a acceptor substrate (Fig. 6b).

For the initial activity assessment, the enzymes were incubated at 37 °C with 3–6 µM purified protein. The sialylation product of LST-a, DSLNT, was readily detected using TLC and HPLC. At a donor-to-acceptor molar ratio of 1, DSLNT yields determined by HPLC ranged from 23.3 to 58.1% after overnight (20 h) incubation, varying with enzyme concentration and with the highest yields observed for Δ70ST6GalNAc6 (Fig. 6a). Repeating the experiment with 3 µg of protein and 1 mM of each substrate, the maximum DSLNT yield reached approximately 50% for ST6GalNAc5 (51.90 ± 0.56) and ST6GalNAc6 (58.10 ± 2.6) after 24 h of incubation (Table S4). It has previously been reported that ST6GalNAc5 and ST6GalNAc6 produced in *E. coli* also catalyze the formation of an isomer of DSLNT, in which the Neu5Ac is α2,6-linked to the internal galactose residue rather than to the GlcNAc unit (Pei et al. 2024). No isomers were detected in the current work (Figure S4).

Using Δ71ST6GalNAc5 and Δ70ST6GalNAc6 at a protein concentration of 2.5 µM demonstrated product build-up over the 20 h of reaction (Fig. 6c, d). Varying the reaction temperature, the strongest TLC spot was observed at 25 and 37 °C. While the Δ71ST6GalNAc5 enzyme was found to be active at both acidic and neutral pH, within a pH range of 5.5 to 7.0, Δ70ST6GalNAc6 showed good activity only at neutral pH (Fig. 6c, d).

Since the ability of ST6GalNAc5 and ST6GalNAc6 to catalyze α−2,6-sialylation on oligosaccharides may depend on the sialylation status of terminal galactose, we tested α−2,6-sialylation on three additional acceptors: LNT and LNTII, which lack sialylation moieties, and 3′-SGNB, which resembles O-glycan structures and the non-reducing end of LST-a, yet with GalNAc in the place of GlcNAc. TLC analysis revealed no detectable products when using these alternative acceptors (Fig. 6c, d).

The apparent *K*_m_ values towards the natural, non-modified form of LST-a were estimated at 37 °C, with Δ71ST6GalNAc5 showing a 4.2-fold lower *K*_m_ than Δ70ST6GalNAc6 (Table 3; FigureS5). Similarly, the turnover number was 1.7-fold higher for Δ71ST6GalNAc5 than for Δ70ST6GalNAc6; hence, its catalytic efficiency was 7.4-fold higher (Table 3).

**Table 3:** Kinetic parameters of recombinant Δ71ST6GalNAc5 and Δ70ST6GalNAc6 towards LST-a (Figure S3)

Enzyme	Production host	Acceptor modification	*K*_m_ (mM)	*k*_cat_ (min^−1^)	*k*_cat_/*K*_m_ (min^−1^ mM^−1^)
ST6GalNAc5	*Pichia pastoris*, this study	-	0.42 ± 0.10	6.29 ± 1.36	15.00 ± 0.29
ST6GalNAc5	HEK293 (Bao et al*.*, 2024)	ProCbz	1.35 ± 0.02	37.65 ± 0.37	27.94 ± 0.56
ST6GalNAc6	*Pichia pastoris*, this study	-	1.79 ± 0.06	3.65 ± 0.24	2.04 ± 0.07
ST6GalNAc6	HEK293 (Bao et al*.*, 2024)	ProCbz	2.94 ± 0.20	159.12 ± 7.42	54.04 ± 3.39

### Enzymatic removal of *N*-glycans

**Fig. 7:**
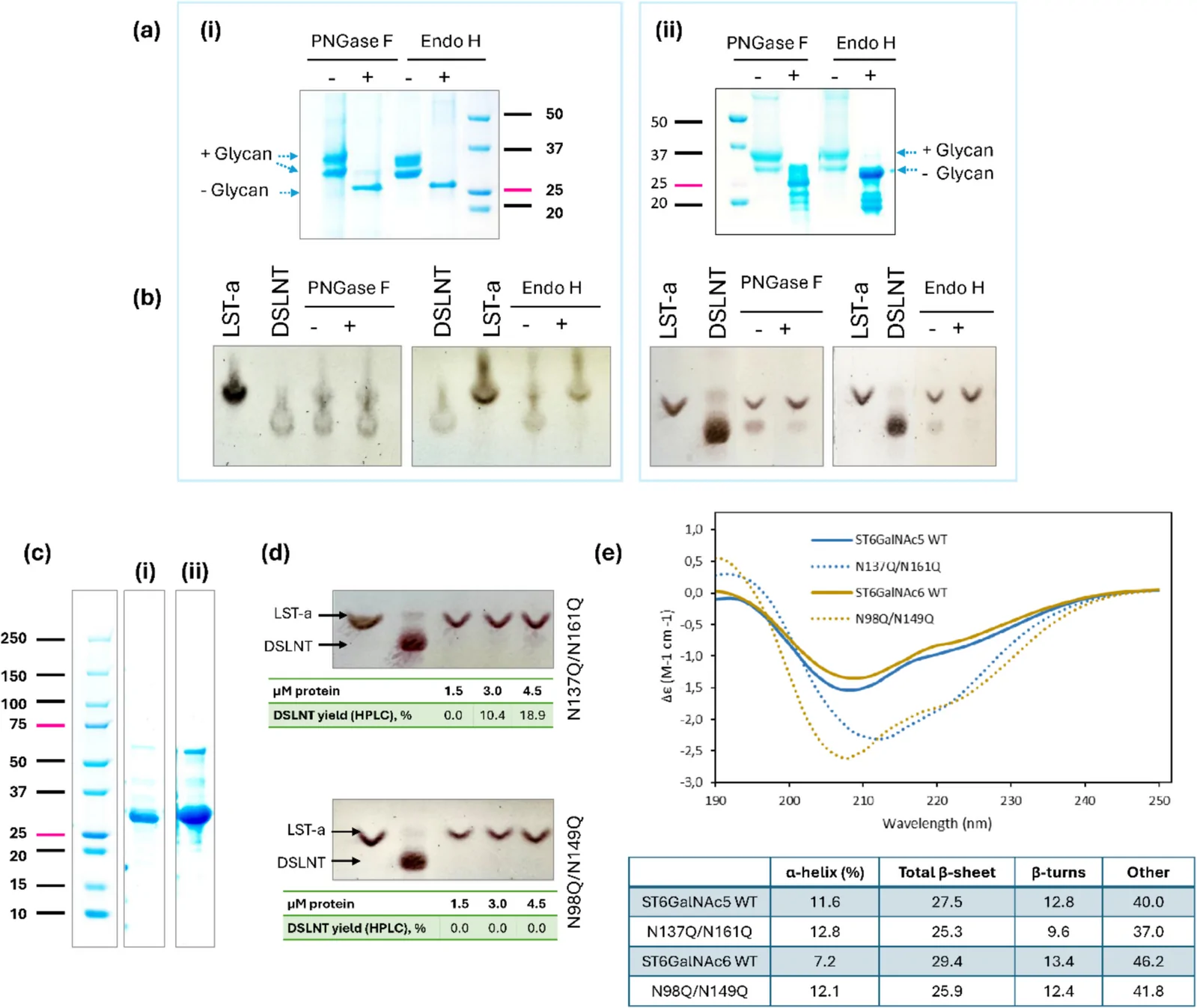
Removal of Δ71ST6GalNac5 and Δ70ST6GalNac6 *N*-glycosylation by enzymatic treatment and site-directed mutagenesis. (**a**) SDS-PAGE gel showing glycosylation of (i) Δ71ST6GalNAc5, and (ii) Δ70ST6GalNAc6 produced in yeast after *N*-glycosylation removal by PNGase F and Endo H, leading to shifts in size. (**b**) Enzymatic activity of reference samples (untreated, -) and samples treated with endoglycosidases (+). (**c**) SDS-PAGE analysis of the purified (i) N137Q/N161Q mutant of ST6GalNAc5 and (ii) the purified N98Q/N149Q mutant of ST6GalNAc6 consistent with the molecular mass of proteins lacking *N*-glycans. (**d**) Functional impact of the double mutations at the *N*-linked glycosylation sites shown by low DSLNT yields evaluated by both TLC and HPAEC. (**e**) CD spectra of two WT and two mutant proteins recorded in the wavelength range 190–250 nm highlighting the subtle change in the content of different types of secondary structures for mutant proteins. Uncropped versions of SDS-PAGE gels are available in Figure S7

Yeast cells are capable of glycosylating proteins but add the core (Man)₈-(GlcNAc)₂ groups only, thus lacking the complex glycans found in mammals. To determine whether the two distinct bands observed for Δ71ST6GalNAc5 and Δ70ST6GalNAc6 on SDS-PAGE and Western blots (Fig. 3; Fig. 4) represent differentially glycosylated forms in our purified enzyme preparation, we treated purified samples with endoglycosidase H (Endo H) and peptide-*N*-glycosidase F (PNGase F), followed by SDS-PAGE analysis (Fig. 7a). Treatment with Endo H, which cleaves high-mannose and some hybrid oligosaccharides, reduced both bands to the predicted molecular weight (~ 32 kDa) (Fig. 7a). Similarly, digestion of ST6GalNAc5 and ST6GalNAc6 with PNGase F, which removes all types of *N*-linked oligosaccharides (Maley et al., 1989), yielded the same result, indicating that *P. pastoris*–expressed ST6GalNAc5 and ST6GalNAc6 contained only high-mannose glycans and lacked complex sugars. Interestingly, the PNGase F–treated sample retained its sialylation activity; the Endo H-treated sample exhibited impaired activity (Fig. 7b). Since the rate of glycan cleavage under native conditions is lower compared to denaturing conditions, according to the manufacturer, the reactions were incubated overnight. Differences in the rate of glycan removal by Endo H and PNGase F, as well as the resulting stability of partially or fully deglycosylated proteins, may explain the observed differences in activity between proteins treated with Endo H and those treated with PNGase F.

### Removal of *N*-linked glycosylation sites by mutagenesis

To further study the effects of glycosylation, we created the Δ71ST6GalNAc5 N137Q/N161Q and Δ70ST6GalNAc6 N98Q/N149Q mutants on a basis of constructs #3 and #4, respectively, and expressed them in *P. pastoris* X-33 cells. Their apparent size on SDS-PAGE was consistent with a lack of *N*-glycosylation (Fig. 7c). The secreted expression levels of both mutant constructs were reduced 1.5-fold compared to the wild-type constructs (TableS5). The activity of the Δ71ST6GalNAc5 N137Q/N161Q and Δ70ST6GalNAc6 N98Q/N149Q variants was assessed using protein concentrations ranging from 1.5 to 4.5 µM at 37 °C and 25 °C, with very low (if any) levels of DSLNT detected by TLC and HPLC (Fig. 7d, Figure S6). At 4.5 µM mutant protein in the reaction solution, the amount of produced DSLNT reached 0.189 ± 0.005 mM, a similar level to that produced with 1.0 µM wild-type protein (0.202 ± 0.036) (Figure S7). In a similar protein concentration range, no product formation was detected for the Δ70ST6GalNAc6 N98Q/N149Q mutant (Fig. 7d).

Glycosylation also influenced the thermal stability of Δ71ST6GalNAc5 and Δ70ST6GalNAc6. Compared to the wild type, the Δ71ST6GalNAc5 N137Q/N161Q mutant exhibited an onset-of-melting temperature reduced by 22 °C (Tris buffer). However, the *T*_m_ values of the Δ71ST6GalNAc5 wild type and mutant were similar (Fig. 5). On the contrary, the N98Q/N149Q mutant of Δ70ST6GalNAc6 reduced both the onset and melting temperatures by 12 °C and 7.3 °C in Tris buffer and by 20 °C and 10 °C in HEPES buffer (Fig. 5). No major differences were observed between Tris and HEPES buffers, except for the onset-of-melting temperature of the Δ70ST6GalNAc6 N98Q/N149Q mutant.

The two mutants also showed clear changes in their CD spectra, indicating conformational changes compared to the wild-type protein. The CD spectra of both the wild-type and mutant proteins at pH 7.0 (Fig. 7e) indicate a mixed α-helical and β-sheet conformation. Analysis of the secondary structure composition revealed that in the Δ71ST6GalNAc5 N137Q/N161Q and Δ70ST6GalNAc6 N98Q/N149Q mutants, the α-helical content slightly increased, primarily due to a reduction in β-sheet elements (Fig. 7e).

## Discussion

To our knowledge, this study is the first to describe the successful expression and purification of active, recombinant human ST6GalNAc5 and ST6GalNAc6 in *Pichia pastoris*. Most research on *P. pastoris* expression has been conducted with Mut^+^ strains such as X-33, which grow faster on methanol and are generally assumed to yield higher recombinant protein levels (Kim et al., 2009). However, several studies have reported superior protein production in Mut^S^ strains such as KM71H under specific conditions (Orman et al., 2009; Ang et al., 2016). In this case, the KM71H/pPICZαA expression system was less effective than the X-33/pPICZαA system in terms of total protein expression and secretion.

The cytoplasmic, transmembrane, and stem regions at the N-terminus of STs contribute to Golgi retention (Banfield, 2011). Previous studies have shown that truncation of the N-terminal stem region in ST6Gal1 enhances expression in *P. pastoris*, improves stability, and facilitates protein crystallization, while maintaining full enzymatic activity (Ribitsch et al., 2014; Moremen et al., 2018). In the current work, we used a truncation strategy based on AlphaFold models, removing all parts of the N-terminal region that appeared disordered in the model. However, direct fusion of this sequence to the α secretion factor resulted in protein secretion levels < 10%. Glycine-serine peptide linkers like (GGGS)₂ are commonly used in protein engineering to introduce flexible and hydrophilic spacers between protein domains (Ceballos-Alcantarilla and Merkx, 2021). In our case, we added the linker to improve the processing of cleavage sites in the α secretion factor and thereby enhance protein secretion.

Our results indicate that, across most tested constructs and conditions, the strategy of including a (GGGS)₂ linker for the heterologous expression of His_6_-tagged ST6GalNAc5 and ST6GalNAc6, combined with lower temperatures in *P. pastoris* X-33 cultures, proved to be more effective for obtaining secreted, active enzymes. While 28 °C is more physiologically suitable for growth, several studies have demonstrated that lower temperatures reduce cell death and protein degradation while improving secretion and heterologous protein expression (Li et al., 2001; Gasser et al., 2007; Dragosits et al., 2009; Zhong et al., 2014), and indeed expression was improved at 20 °C as compared to 28 °C in the current work. The total amount of produced protein did not exceed 7 mg/L of cell culture under any of the initially tested conditions, with approx. 3 mg/L being the maximum of secreted protein. While these values are lower than the typically reported levels of other eukaryotic proteins, protein expression in *P. pastoris* is often target-specific and requires an individualized optimization approach.

In general, protein expression and secretion can be enhanced by optimizing media components. Various additives have been shown to improve protein yields in *P. pastoris* (Mengwasser et al. 2011), with mixed carbon sources such as low levels of glucose, glycerol, and sorbitol enhancing expression (Xie et al., 2003; Jungo et al. 2007; Paulová et al. 2012). However, the addition of glycerol did not improve expression in this case.

The production of recombinant eukaryotic proteins in yeast, even at relatively low expression levels, can impose a metabolic burden and cause cellular stress due to post-translational processes such as protein folding and secretion, thereby compromising their overall growth and protein production rates (Glick, 1995; Mattanovich et al., 2004; Ramon et al., 2007). This includes the degradation of misfolded or unfolded proteins in the endoplasmic reticulum and inefficient or unsuitable secretion (Mattanovich et al., 2004; Cos et al., 2005; Resina et al., 2007; Gasser et al., 2008). In addition to protein degradation, increased energetic demands related to the cost of the folding, refolding, and secretion processes of the protein product could result in an overall readjustment of metabolism, including that related to amino acids (Carnicer et al., 2012). As such, magnesium and amino acids are gradually depleted in the expression media, and this can potentially limit recombinant protein production by *P. pastoris*. Amino acid supplementation has been shown to help alleviate the burden on cellular metabolism (Heyland et al. 2011) and to have a positive effect on recombinant protein production (Görgens et al. 2004; Hahn-Hägerdal et al. 2005; Heyland et al. 2010). The improved expression of secreted, active enzyme achieved in the current work upon media supplementation with Casamino acids and MgCl_2_ suggests that the observed limitation in recombinant protein production is not due to constraints in transcription or translation machinery but rather stems from metabolic limitations.

Under all tested conditions, the secretion efficiency did not exceed 60% at 20 °C, indicating that a large proportion of the produced protein remained in the cell pellets. In mammalian cells and the baculovirus-insect cell system previously used for ST protein expression, overall expression levels (cells + media) typically exceed 10 mg/L, though secretion efficiency varies (Moremen et al., 2018). Notably, Moremen et al. (2018) reported secretion levels of ≥ 40 mg/L in HEK293 cells for ST6GalNAc5 (residues 50–336, N-terminal GFP fusion) and ST6GalNAc6 (residues 31–333, N-terminal GFP fusion). However, their level of secretion was comparable to our results, namely around 50%. It appears that in *P. pastoris* shake flask expression, the current expression limit for active ST6GalNAc5 and ST6GalNAc6 is 10–15 mg/L with potential for further optimization.

As an optimization step towards increased expression of ST6GalNAc5 and ST6GalNAc6 in *P. pastoris*, the use of fusion proteins, such as a His₆-SUMO fusion tag, can be considered, especially since it has already been shown to work with flexible linkers similar to the one we used (Zhan et al., 2021; Li et al., 2024). To improve the number of properly folded proteins, co-expression of chaperones involved in protein folding within the endoplasmic reticulum (ER), such as PDI1, ERO1, and HAC1, can be considered (Liu et al., 2023). The addition of protease inhibitors is commonly used to avoid protein degradation. In addition, aside from the inducible AOX1 promoter used in this study, constitutive promoters such as GAP and PMA could also be tested for driving target gene expression in *Pichia* (Erden-Karaoğlan and Karaoğlan 2022).

Functional expression is crucial for evaluating recombinant protein success. While engineered *E. coli* strains optimized for disulfide bond formation were recently shown to produce ST6GalNAc5 and ST6GalNAc6, the first results on recombinant expression of these two enzymes in *E. coli* exhibited significantly lower sialylation activity on LST-a than HEK293-derived enzymes, as well as formation of significant amounts of an undesirable DSLNT isomer (Pei et al. 2024; Bao et al., 2024). We confirmed robust sialylation activity in *P. pastoris* X-33-derived ST6GalNAc5 and ST6GalNAc6 and no formation of undesired isomers.

Maximum DSLNT yields of 52% and 58% were obtained for Δ71ST6GalNAc5 and Δ70ST6GalNAc6, respectively, with 1 mM of each substrate. Thus, the difference between the two enzymes was less pronounced than observed for HEK293-produced ST6GalNAc5 and ST6GalNAc6, which showed similar and higher conversion rates of LST-a, which was modified at the reducing end by attachment of a 2-*O*-(*N*-benzyloxycarbonyl)aminopropyl (ProCbz) group (LST-a-ProCbz) at 20 mM CMP-Neu5Ac (53.1% and 99%, respectively, after overnight incubation) (Bao et al., 2024). In the case of HEK293-produced enzymes, ST6GalNAc6 was the more effective enzyme, showing not only higher conversion rates but also a twofold greater *k*_cat_/*K*_m_ value than ST6GalNAc5 (Bao et al., 2024). The apparent *K*_m_ values exhibited by the recombinant yeast-produced ST6GalNAc5 and ST6GalNAc6 towards the natural, non-modified form of LST-a at 37 °C were 1.6 to 3.2 times lower than those of the HEK293-produced enzymes towards LST-a-ProCbz (Bao et al., 2024). In our study, yeast-produced Δ71ST6GalNAc5 was 7.4-fold more efficient (based on the *k*_cat_/*K*_m_ ratio) in the sialylation assay than Δ70ST6GalNAc6. While the catalytic efficiency of yeast-produced ST6GalNAc5 was reduced by only 1.9-fold compared to the HEK293-produced enzyme, a significantly greater reduction was observed between the two ST6GalNAc6 samples. In contrast to recent studies, which reported the activity of HEK293-produced GFP-fused ST6GalNAc5 and ST6GalNAc6, as well as of *E. coli*-expressed MBP-fused ST6GalNAc5, towards several ProCbz-modified acceptors, including ProCbz-modified LNT (Bao et al., 2024; Bai et al., 2025), we did not observe activity on other acceptors alternative to LST-a.

Glycosylation is a common post-translational modification in eukaryotic expression systems, and *N*-glycosylation occurs on asparagine (Asn) residues within N-X-S/T motifs and influences protein folding, signaling, trafficking, cell–cell interactions, and immune responses (Dwek, 1998; Rudd et al., 2001; Brennan et al., 2011). However, its role in STs remains largely unexplored. We observed an important dependence of enzymatic activity on glycosylation status. PNGase F and Endo H treatment to remove *N*-linked glycans confirmed the glycosylation of ST6GalNAc5 and ST6GalNAc6 in *P. pastoris* X-33 expression systems. Protein sequence analysis and site-directed mutagenesis identified two asparagine residues in each protein that are essential for *N*-linked glycosylation and, consequently, for enzymatic stability and activity in vitro. By site-directed mutagenesis, we substituted the Asn residues at the two predicted *N*-glycosylation sites were substituted with Gln residues to prevent glycosylation while preserving the polar carboxamide side chain, thereby minimally affecting the tertiary protein structure by this mutation. The removal of *N*-linked glycosylation sites through mutagenesis may result in reduced activity compared to glycosylated proteins (Branza-Nichita et al., 2000). While the removal of these bulky, flexible groups is generally thought to facilitate expression and crystallization, the impact of *N*-glycosylation must be assessed for each individual protein. In the case of Δ71ST6GalNAc5 N137Q/N161Q and Δ70ST6GalNAc6 N98Q/N149Q, the resulting loss of activity was severe. Recently, the recombinant expression of hST6GALNAC5 in *E. coli*, which does not form *N*-glycosylations during expression, demonstrated that the addition of an N-terminal MBP domain may be an alternative means of conferring ST stability and activity during recombinant expression (Bai et al., 2025).

Analysis of enzyme thermal stability indicated that the two recombinantly produced enzymes were equally stable and may be used in vitro at slightly higher temperatures than their natural, physiological temperature of 37 °C. ST6GalNAc5 and ST6GalNAc6 have very similar overall structures, characterized by an α/β protein scaffold. Mutations that alter *N*-glycosylation can significantly impact protein activity in vitro, as *N*-glycans contribute to stabilizing proper protein conformation (Nagai et al., 1997; Kitazume-Kawaguchi et al., 1999; Shauchuk et al. 2020). Consequently, the removal of glycans from ST proteins can destabilize them by increasing their susceptibility to proteolysis and/or enhancing their tendency for self-association (Chen and Colley, 2000). Indeed, removal of the conserved *N*-glycosylation sites common within the ST6GALNAC subfamily in the N137Q/N161Q and N98Q/N149Q mutants led to reduced thermal stability and detectable changes in secondary structure content, as observed by NanoDSF and CD analysis. These findings suggest that the conserved *N*-glycosylation sites play a crucial role in maintaining the conformation of ST6GALNAC subfamily enzymes.

## Supplementary Information

Below is the link to the electronic supplementary material.

ESM 1 (DOCX 999 KB)

## Data Availability

The two codon-optimized DNA sequences used for protein expression have been submitted to NCBI GenBank with accession numbers PV837576 and PV837577. All data generated or analyzed during this study are included in this published article and its supplementary information files.
