# Supplementary material for: Expression of functional human sialyltransferases ST6GalNAc5 and ST6GalNAc6 in Pichia pastoris
Source: Appl Microbiol Biotechnol. 2025 Oct 15;109(1):222. doi: 10.1007/s00253-025-13607-x (PMC12521282; doi:10.1007/s00253-025-13607-x)
Supplement: Supplementary file 1 — (DOCX 999 KB) [file 253_2025_13607_MOESM1_ESM.pdf]

Supplementary Information for

Expression of functional human sialyltransferases ST6GalNAc5 and ST6GalNAc6 in *Pichia pastoris*

Ganna Krasnoselska[1], Marton Lengyel[2], Martin Matwiejuk[2], Marlene Vuillemin[1], Dora Molnar-Gabor[2], Anne S. Meyer[1], Birgitte Zeuner[1]*

[1]Department of Biotechnology and Biomedicine, Technical University of Denmark, Søltofts Plads, 2800 Kgs. Lyngby, Denmark
[2]DSM-Firmenich, Kogle Allé 4, 2970 Hørsholm, Denmark.
*Corresponding author: bzeu@dtu.dk

Table S1. DNA sequences of Δ71ST6GalNAc5 and Δ70ST6GalNAc6 codon-optimized for expression in *Pichia pastoris*.

**Δ71ST6GalNAc5**
GCTGGTCCAAGACCTTTGGATGGTTACTTGGGTGTTGCTGATCATAAGCCATTGAAAATGCACTGTAGA<br>GATTGTGCTTTGGTTACTTCTTCTGGTCATTTGTTGCACTCTAGACAAGGTTCTCAAATCGATCAAACT<br>GAATGTGTTATCAGAATGAACGATGCTCCTACTAGAGGTTATGGTAGAGATGTTGGTAATAGAACTTCT<br>TTGAGAGTTATTGCTCATTCTTCTATCCAAAGAATTTTGAGAAACAGACACGATTTGTTGAACGTTTCT<br>CAAGGTACTGTTTTTATTTTCTGGGGTCCTTCTTCTTACATGAGAAGAGATGGTAAAGGTCAAGTTTAC<br>AACAATTTGCATTTGTTGTCTCAAGTTTTGCCAAGATTGAAGGCTTTCATGATCACTAGACACAAGATG<br>TTGCAATTCGATGAATTGTTCAAACAAGAGACTGGTAAAGATAGAAAGATCTCTAACACTTGGTTGTCT<br>ACTGGTTGGTTTACTATGACTATTGCTTTGGAGTTGTGTGATAGAATTAACGTTTACGGTATGGTTCCA<br>CCTGATTTCTGTAGAGATCCAAATCATCCTTCTGTTCCATACCACTACTATGAACCTTTTGGTCCAGAT<br>GAGTGTACTATGTATTTGTCTCATGAAAGAGGTAGAAAAGGTTCTCATCACAGATTCATTACTGAGAAG<br>AGAGTTTTCAAAAACTGGGCTAGAACTTTTAATATCCATTTCTTTCAACCTGATTGGAAGCCAGAATCT<br>TTGGCTATTAACCACCCTGAGAACAAGCCAGTTTTC
**Δ70ST6GalNAc6**
TACGGTTCTTTGCGAGGACGAAGCCGCCGTCCGGTAAATCTGAAAAAATGGAGTATAACTGACGGTTAT<br>GTTCCCATACTAGGCAACAAGACATTACCTTCTCGTTGCCATCAGTGCGTGATAGTGAGTTCCTCCAGT<br>CACCTTTTGGGCACGAAGTTGGGTCCTGAAATTGAGAGGGCGGAGTGTACAATTAGAATGAATGACGCT<br>CCAACTACGGGGTACTCTGCAGATGTGGGCAACAAGACTACGTATAGAGTCGTCGCCCATAGCAGCGTA<br>TTTCGTGTACTTAGGCGTCCACAGGAATTTGTGAACCGGACCCCCGAGACCGTATTCATCTTCTGGGGT<br>CCTCCAAGTAAGATGCAGAAACCGCAAGGAAGCCTCGTCCGGGTGATACAGCGGGCCGGTCTGGTCTTC<br>CCTAACATGGAGGCATACGCGGTATCGCCGGGGCGGATGAGACAATTTGATGACTTATTCAGGGGGGAG<br>ACCGGAAAGGACAGAGAAAAATCACATTCATGGCTCAGTACGGGTTGGTTTACCATGGTCATCGCCGTT<br>GAACTATGTGATCATGTTCACGTTTACGGTATGGTGCCACCGAATTACTGTTCTCAAAGGCCGCGACTC<br>CAGCGAATGCCATATCATTATTATGAACCCAAAGGGCCCGATGAATGCGTTACGTACATCCAAAATGAG<br>CACTCGCGCAAAGGAAACCATCACCGCTTCATTACAGAGAAGCGCGTTTTCTCGTCCTGGGCTCAACTG<br>TATGGGATCACTTTTTCACACCCCTCATGGACATGAGTCGAC

Table S2. Set of primers used for the construction of mutants, with forward and reverse primers denoted by "_f" and "_r", respectively. Mutated codons are underlined.

Enzyme template	Primer	Sequence (5' to 3')
Δ71ST6GalNAc5	1: N137Q_f	CTAGAGGTTATGGTAGAGATGTTGGTCAGAGAACTTCTTTGAGAGTTATTG
	2: N137Q_r	CAATAACTCTCAAAGAAGTTCTCTGACCAACATCTCTACCATAACCTCTAG
Δ71ST6GalNAc5 N137Q	3: N161Q_f	CAGACACGATTTGTTGCAAGTTTCTCAAGGTACTG
	4: N161Q_r	CAGTACCTTGAGAAACTTGCAACAAATCGTGTCTG
Δ70ST6GalNAc6	5: N98Q_f	GTTATGTTCCCATACTAGGCCAAAAGACATTACCTTCTCGTTG
	6: N98Q_r	CAACGAGAAGGTAATGTCTTTTGGCCTAGTATGGGAACATAAC
Δ70ST6GalNAc6 N98Q	7: N149Q_f	GTACTCTGCAGATGTGGGCCAGAAGACTACGTATAGAGTCG
	8: N149Q_r	CGACTCTATACGTAGTCTTCTGGCCCACATCTGCAGAGTAC

Table S3. Protein yields from expression at 20°C using methanol alone and methanol supplemented with 0.1% (v/v) glycerol as carbon sources.

Host/construct	Carbon source	Secreted, mg/L	Total, mg/L	Secretion, %
X-33/#3	Methanol	2.5	5.5	45.5
X-33/#3	Methanol + glycerol	0.3	1.3	23.1
X-33/#5	Methanol	2.8	6.0	46.7
X-33/#5	Methanol + glycerol	0.3	3.7	7.0
KM71H/#5	Methanol	3.2	6.4	50.0
KM71H/#5	Methanol + glycerol	0.4	4.8	7.3

Table S4. DSLNT molar yield (%) after 24 h of reaction with 1 mM LST-a, 1 mM CMP-Neu5Ac, and 3 μg of purified protein determined by HPLC-CAD.

Protein	DSLNT yield, %
ST6GalNAc5	51.90 ± 0.56
ST6GalNAc6	58.10 ± 2.6

Table S5. Expression yields and protein secretion of *N*-glycosylation variants Δ71ST6GalNAc5 N137Q/N161Q and Δ70ST6GalNAc6 N98Q/N149Q without supplementation, i.e. at conditions corresponding to Set0 in Table 2.

	ST6GalNAc5 N137Q/N161Q	ST6GalNAc6 N98Q/N149Q
Secreted (mg/L)	1.8 ± 0.2	1.3 ± 0.1
Total protein (mg/L)	6.8 ± 0.4	3.1 ± 0.2
Secretion (%)	25.9 ± 0.9	41.6 ± 0.7
Activity (TLC)	+	n/d

(a)

> sp |Q9BVH7| ST6GalNAc5 1-336:
MKTLMRHGLAVCLALTTMCTSLLLVYSSLGGQKERPPQQQQQQQQQQQAS
ATGSSQPAAESSTQQRPGVPAGPRPLDGYLGVADHKPLKMHCRDCALVTSSGH
LLHSRQGSQIDQTECVIRMNDAPTRGYGRDVGNRTSLRVIAHSSIQRILRNRHD
LLNVSQGTVFIFWGPSSYMRRDGKGQVYNNLHLLSQVLPRLKAFMITRHKMLQ
FDELFKQETGKDRKISNTWLSTGWFTMTIALELCDRINVYGMVPPDFCRDPNHP
SVPYHYYEPFGPDECTMYLSHERGRKGSHHRFITEKRVFKNWARTFNIHFFQPD
WKPESLAINHPENKPVF

> sp |Q969X2| ST6GalNAc6 1-333:
MACSRPPSQCEPTSLPPGPPAGRRHLPLSRRRREMSSNKEQRSAVFVILFALITIL
ILYSSNSANEVFHYGSLRGRSRRPVNLKKWSITDGYVPILGNKTLPSRCHQCVIV
SSSSHLLGTKLGPEIERAECTIRMNDAPTTGYSADVGNKTTYRVVAHSSVFRVLR
RPQEFVNRTPETVFIFWGPPSKMQKPQGSLVRVIQRAGLVFPNMEAYAVSPGRM
RQFDDLFRGETGKDREKSHSWLSTGWFTMVIAVELCDHVHVYGMVPPNYCSQ
RPRLQRMPYHYYEPKGPDECVTYIQNEHSRKGNHHRFITEKRVFSSWAQLYGIT
FSHPSWT

(b)

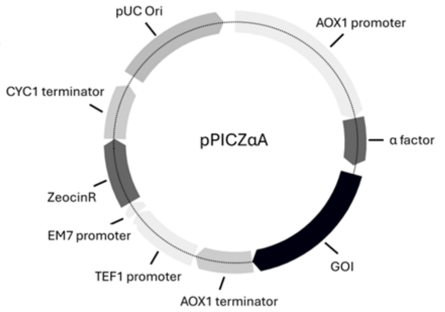

(c)

ST6GalNAc1	360 LRCITCAVV	386 HDYVF	531 QLCDQVSAYGFITEGHER-	559 KRLIF
ST6GalNAc2	146 PKCIRCAVV	172 HDYVF	315 HTCDQVSAYGFITSNYWK-	343 KPLIF
ST6GalNAc3	075 LDCDLCAIV	101 SSCIW	227 DACYGIHVYGMINDTYCKT	261 DECDE
ST6GalNAc4	071 EPCRSCAVV	097 AECVF	223 ELCEEIVVYGMVSDSYCRE	258 DECQM
ST6GalNAc5	091 MHCRDCALV	117 TECVI	243 ELCDRINVYGMVPPDFCRD	278 DECTM
ST6GalNAc6	103 SRCHQCVIV	129 AECTI	254 ELCDHVHVYGMVPPNYCSQ	290 DECVT

(d)

Element	AA in hST6GalNAc5	AA in hST6GalNAc6
Topological (cytoplasmic)	1-6	1-42
Transmembrane (TM)	7-29	43-60
Topological (luminal)	30-336	61-333
polyQ region	38-48	x
pLDDT < 50 (AF2 models)	26-71<br>328-334	1-25<br>63-77
Disulfide bonds	C93 → C119<br>C96 → C245<br>C259 → C280	C105 → C131<br>C108 → C256<br>C270 → C292
N-glycosylation motifs (NXS/T)	N^{137}RT<br>N^{161}VS	N^{98}KT<br>N^{149}KT<br>N^{173}RT

Figure S1: Analysis of the amino acid sequences of human ST6GalNAc5 and ST6GalNAc6, and the design of constructs for expression in yeast. (a) Full-length amino acid sequences of human ST6GalNAc5 and ST6GalNAc6. Conserved cysteines are highlighted in red, and *N*-glycosylation sites are boxed in magenta. Sialylmotifs L are underscored. The catalytic domain of all eukaryotic STs is characterized by four conserved peptide sequences - known as the sialylmotifs large (L), small (S), 3rd (III), and very small (VS) - with residues that play distinct roles in donor and acceptor substrate binding as well as in the catalytic reaction. **(b)** Schematic illustration of the pPICZαA vector used for the expression of target genes (GOI) in *Pichia pastoris*. **(c)** Distribution of cysteine residues in other members of the ST6GalNAc family related to ST6GalNAc5 and ST6GalNAc6. **(d)** Distribution of secondary structure elements in the protein sequences of human ST6GalNAc5 and ST6GalNAc6, based on UniProt data and observations from AlphaFold2 structure models.

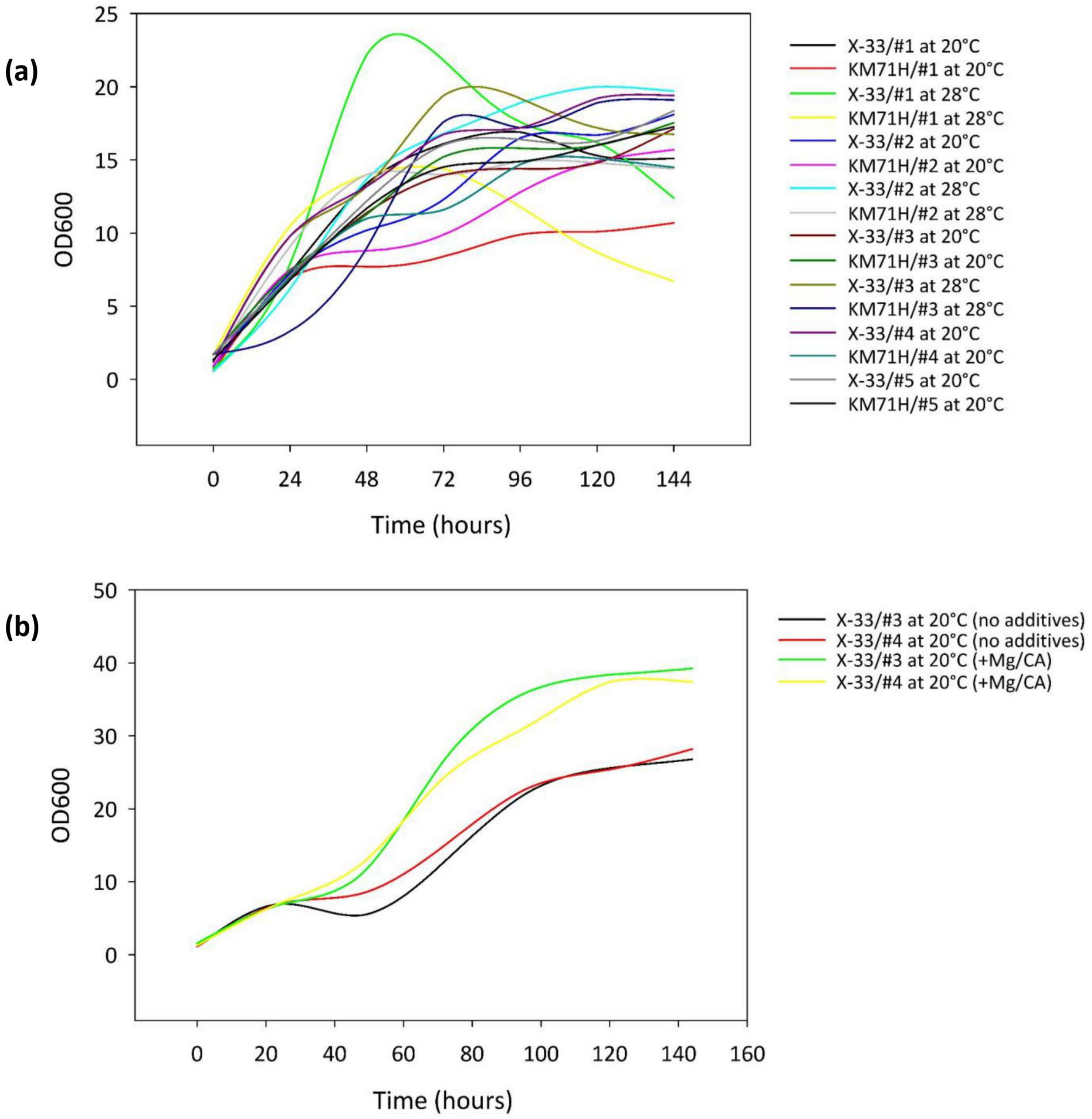

Figure S2: Time courses of cell growth during methanol induction. (a) Cell growth of *Pichia pastoris* X-33 and KM71H transformants at two temperatures with daily addition of 0.5% methanol. Methanol induction was initiated at an OD_{600} of ~1.5 for cells in BMMY media and terminated after 144 hours. **(b)** Cell growth of *Pichia pastoris* X-33 transformants under optimized conditions with addition of $MgCl_2$ and Casamino acids.

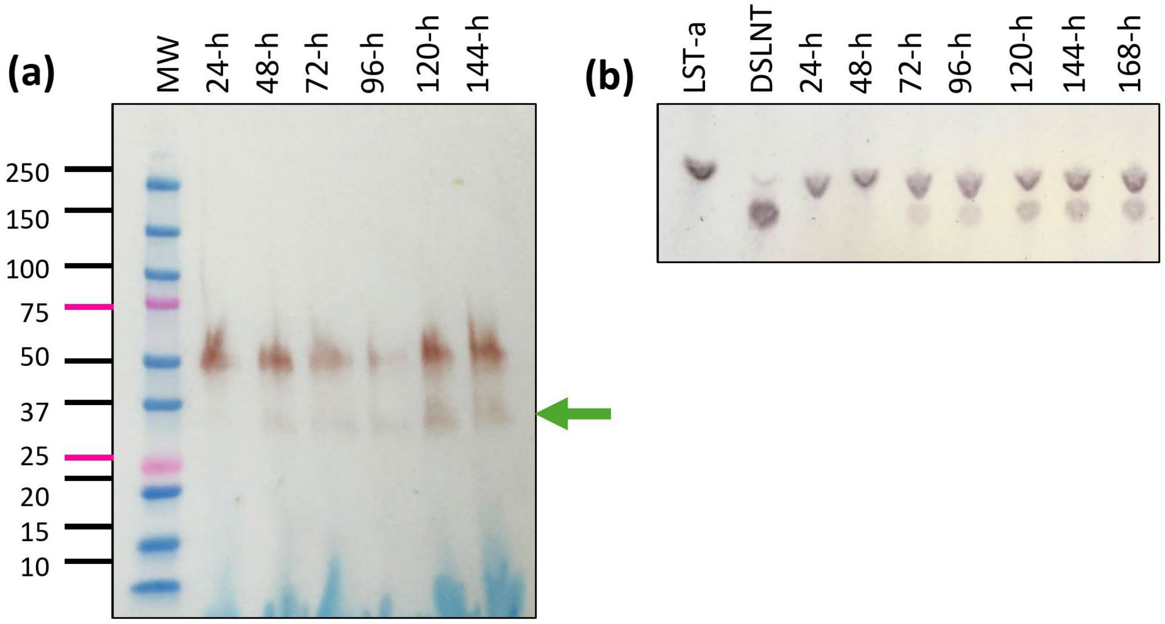

(c)

		Secreted (mg/L)		
**Protein**	Construct	120-h	144-h	168-h
**ST6GalNAc5**	#3	1.8 ± 0.1	2.7 ± 0.2	2.8 ± 0.2
**ST6GalNAc6**	#4	1.4 ± 0.0	1.9 ± 0.2	1.6 ± 0.1

Figure S3: Time progress of ST6GalNAc5 and ST6GalNAc6 expression in *Pichia pastoris* X-33. **(a)** Western blot showing residual (non-secreted) ST6GalNAc5 protein in the cell pellets of *Pichia pastoris* X-33 at various time points following methanol induction. From 48 hours up to 144 hours post-induction, a portion of the produced protein remains within the cells (green arrow indicates the predicted molecular weight of ST6GalNAc5, ~32 kDa). **(b)** TLC plate showing the sialylation activity of ST6GalNAc5 in the culture supernatant collected at different time points after methanol induction. Protein secretion into the medium is observed starting at 72 hours post-induction, with active protein accumulating in the supernatant during the remaining induction period. **(c)** Protein (secreted) yields after harvesting supernatants at 120, 144, and 168 hours post-induction.

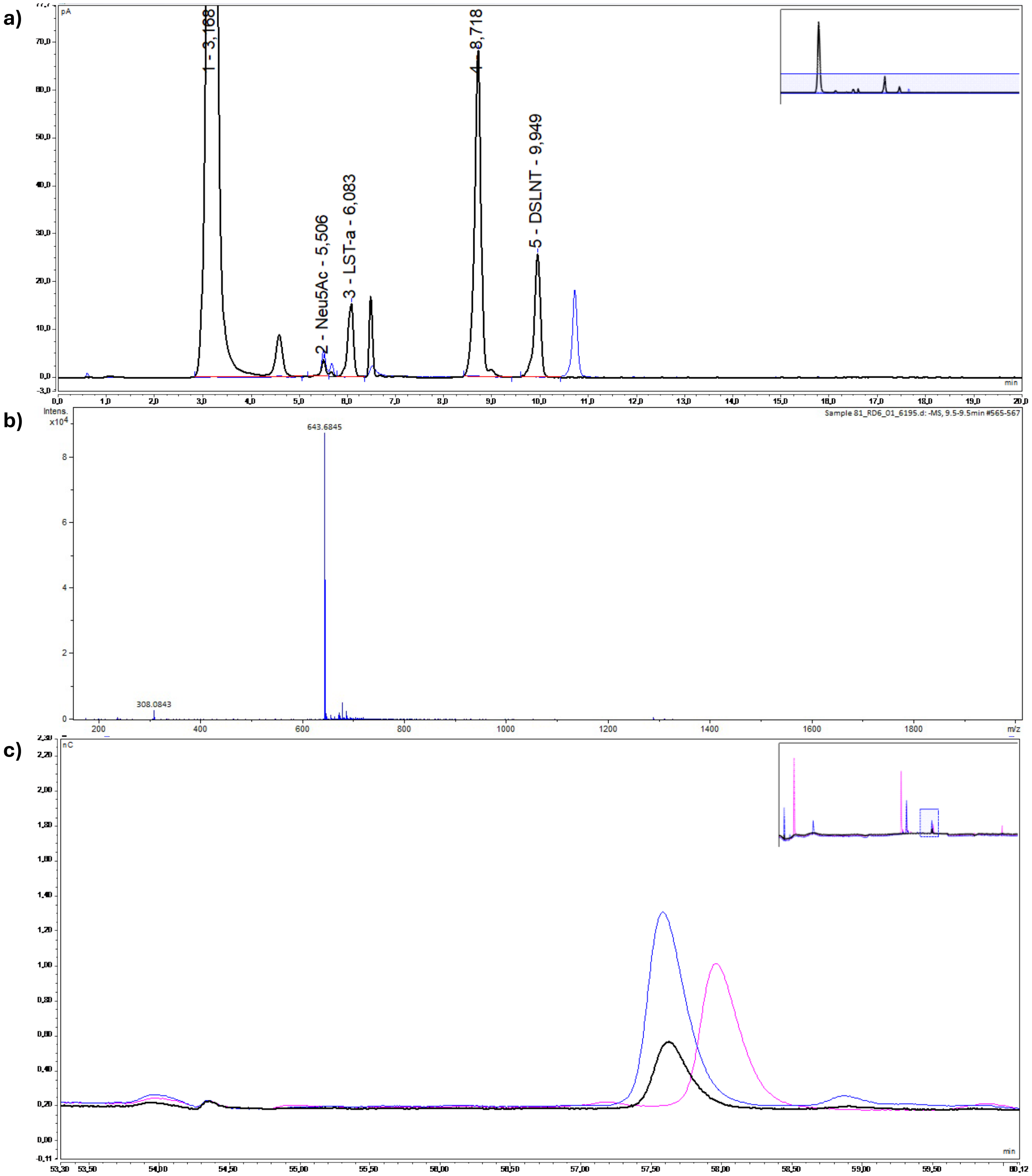

Figure S4: Analysis of DSLNT. (a) Chromatogram from the HPLC-CAD analysis of a reaction mixture with CMP-Neu5Ac conversion (black) and CMP-Neu5Ac reference (blue). The peak at 8.718 min is sodium, and the peak at 3.168 min is another component of the reaction buffer. **(b)** Mass spectrum of the DSLNT peak in the reaction mixture. **(c)** Overlaid HPAEC-PAD chromatograms of the external DSLNT standard (black), a reaction sample (blue), and a DSLNT isomer (pink) prepared as a transsialylation reaction between 6'SL and LST-a catalyzed by a bacterial GT80 α2,6-sialyltransferase known to sialylate O-6 of the terminal and internal galactose units.

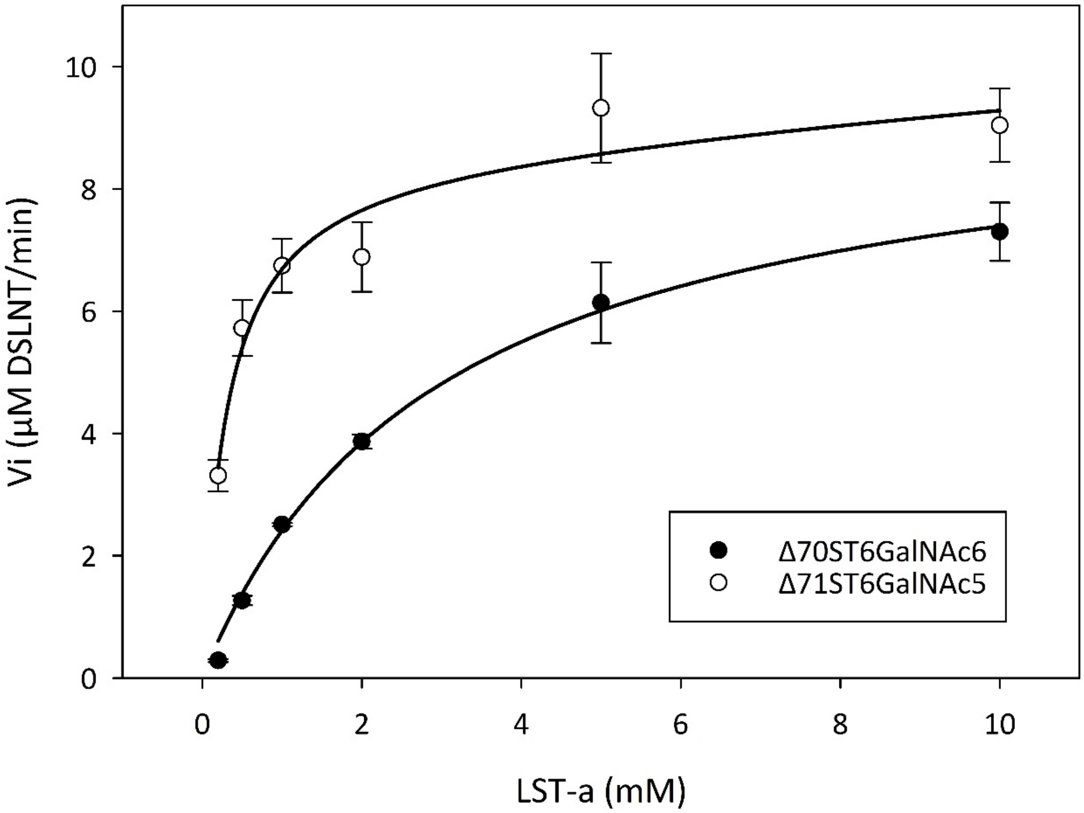

Figure S5. Michaelis-Menten kinetics of the enzymatic activity of purified wild-type Δ71ST6GalNAc5 and Δ70ST6GalNAc6 toward LST-a. The enzymatic reaction rate is shown as the relationship between the concentration of the LST-a substrate and the initial rate of DSLNT product formation.

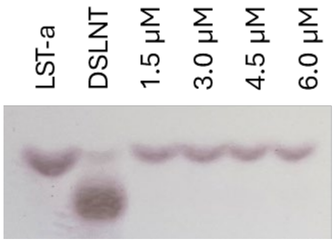

Figure S6. TLC plate showing no reaction products after overnight incubation at 25 °C with different amounts of purified ST6GalNAc5 N137Q/N161Q, 1 mM LST-a, and 1 mM CMP-Neu5Ac.

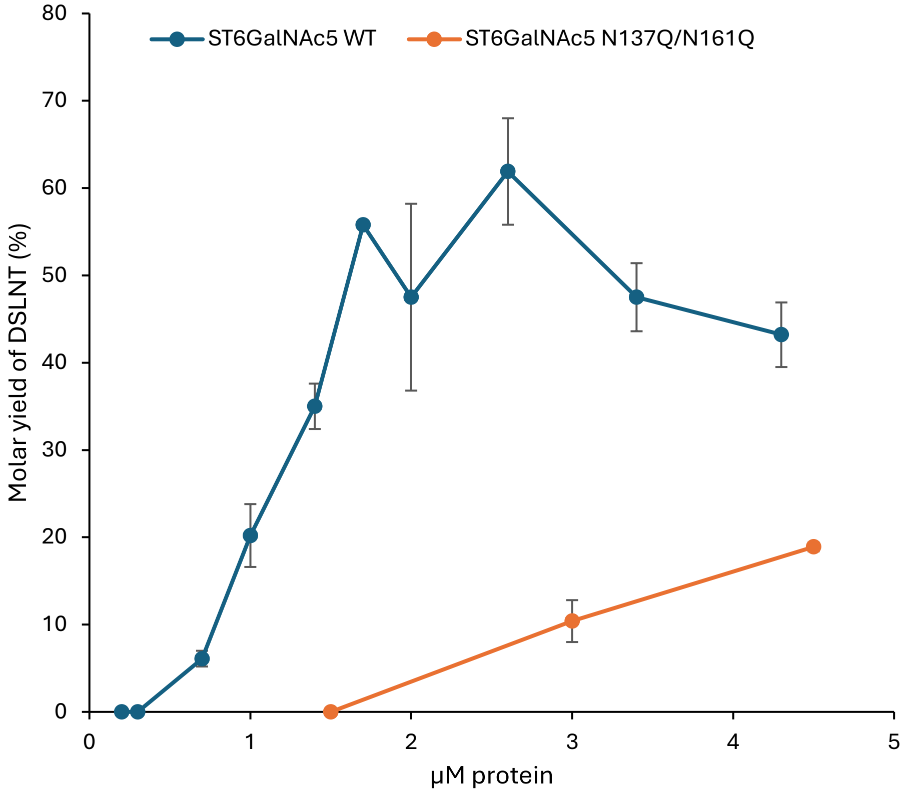

Figure S7. DSLNT molar yield (%) formed by ST6GalNAc5 WT (blue) and N137Q/N161Q (orange) after overnight reaction with 1 mM LST-a, 1 mM CMP-Neu5Ac, and different amounts of purified protein.

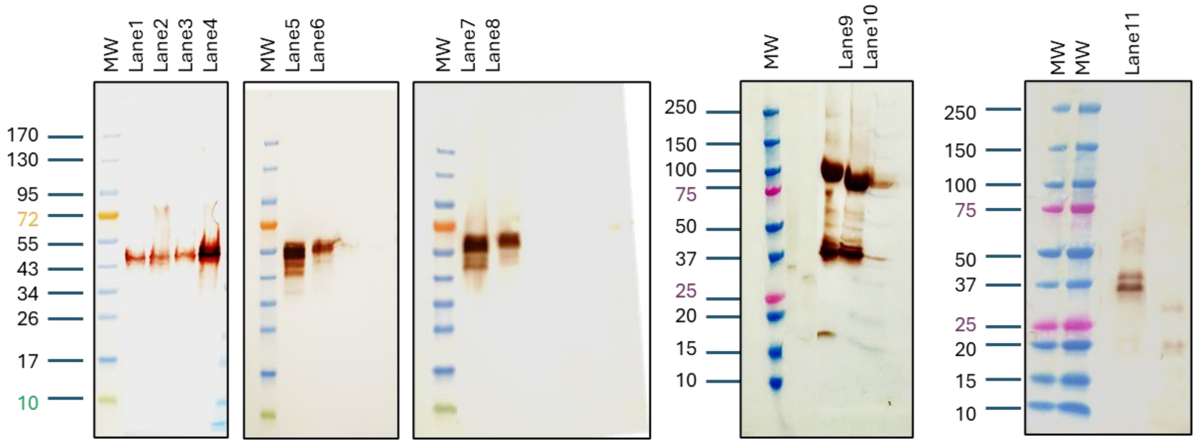

Figure S8. Uncropped Western blot (WB) gels. The gel lanes corresponding to the cropped areas in the indicated images are shown. Lanes 1–10 correspond to N1, N2, N5, N6, N9, N10, N13, N14, N17, and N18, respectively, in Figure 2b. Lane 11 corresponds to #3a in Figure 4.

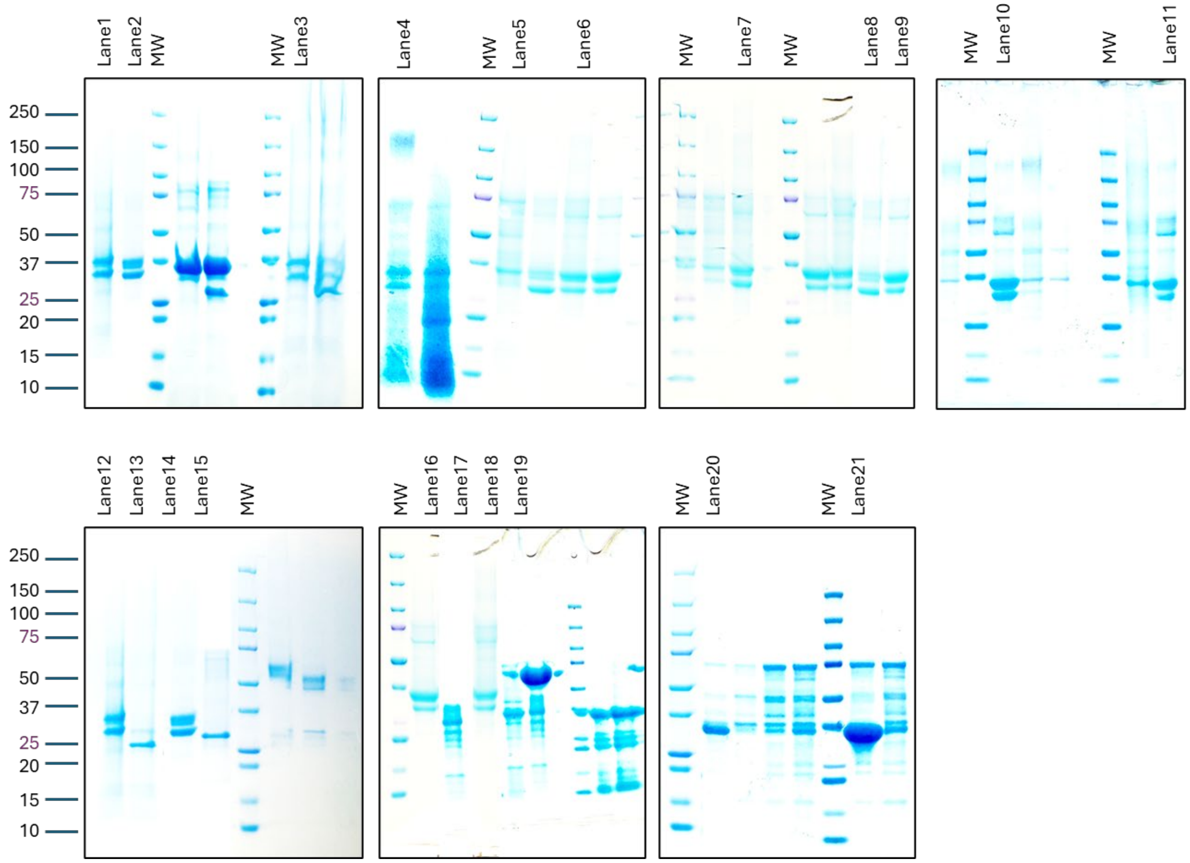

Figure S9. Uncropped SDS-PAGE gels. The gel lanes corresponding to the cropped areas in the indicated images are shown. Lanes 1–6 correspond to Sets 0, 1, 2, 3, 4, and 5 for ST6GalNAc5 WT in Figure 3a. Lanes 7–11 correspond to Sets 0, 4, 5, 1, and 6 for ST6GalNAc6 WT in Figure 3a. Lanes 12–15 correspond to PNGase F '−', PNGase F '+', Endo H '−', and Endo H '+' in Figure 6a, panel (i). Lanes 16–19 correspond to PNGase F '−', PNGase F '+', Endo H '−', and Endo H '+' in Figure 6a, panel (ii). Lanes 20 and 21 correspond to panels (i) and (ii) in Figure 6c.
